# Functional Morphology of the Inner Ear of Southern Bluefin Tuna, *Thunnus maccoyii* (Scombridae, Teleostei): Adaptations for Hearing Within a Pelagic Environment

**DOI:** 10.1002/cne.70220

**Published:** 2026-11-04

**Authors:** Myoung‐Hoon Ha, Lucille Chapuis, Travis L. Dutka, Julian Pepperell, Caroline C. Kerr, Rebecca Glarin, Bradford Moffat, Shaun P. Collin

**Affiliations:** ^1^ School of Agriculture, Biomedicine and Environment La Trobe University Melbourne Victoria Australia; ^2^ Pepperell Research & Consulting Pty Ltd Doonan Queensland Australia; ^3^ Max Planck Queensland Centre (MPQC) for the Materials Science of Extracellular Matrices Queensland University of Technology Kelvin Grove Queensland Australia; ^4^ Melbourne Brain Centre Imaging Unit, Department of Radiology, Medicine, Dentistry and Health Sciences University of Melbourne Melbourne Victoria Australia

**Keywords:** audition, auditory nerve, hair cell density, otolith, otolithic end organ, pelagic teleost, scanning electron microscopy

## Abstract

The inner ears of teleosts are structurally diverse, reflecting the crucial role of audition in sound detection, balance, and social communication within each species’ ecological niche. However, very little is known about the inner ear morphology of large pelagic teleosts. This study investigates the functional morphology of the inner ears of southern bluefin tuna *Thunnus maccoyii*, using scanning electron microscopy and magnetic resonance imaging, and examines the auditory sensory epithelia (maculae) of the three otolithic end organs (saccule, lagena, and utricle). The results show the inner ears of *T. maccoyii* are enveloped in compact otic capsules, cartilage‐like semicircular canals, and an interaural angle (35° from the midline) between the left and right saccules. The hair cell types are found to be heterogeneously distributed, where hair cell types with longer kinocilia lie along the periphery of the maculae, while the hair cell type with shorter kinocilium lies along the center of the maculae. Six groups of hair cells with opposing orientations are found in the saccular macula, where each opposing orientation group is innervated by separate rami of the VIIIth cranial nerve. The mean hair cell densities of the saccule, lagena, and utricle are 20,400, 39,300, and 36,400 mm^−2^, respectively. The lagenar and utricular maculae are only partially covered by the overlying otolith. These findings contribute to the understanding of morphological adaptations of inner ears in pelagic teleosts and highlight the need for further research on structure‐function relationships in the auditory system of these species.

## Graphical abstract

In this article, we provide the first extensive neuroanatomical characterization of the inner ear of southern bluefin tuna, using scanning electron microscopy. Morphological results include six distinct hair cell orientation groups in the saccule, partial otolith coverage in the lagena and the utricle, and presence of a high density of hair cells with a long kinocilium in the macular areas not covered by the otoliths.

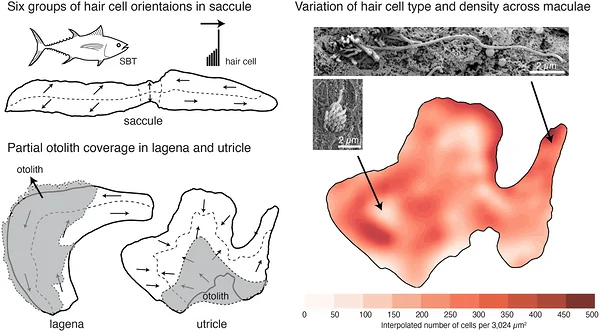

## 1 Introduction

The tunas within the genus *Thunnus*, of which there are eight species (Collette et al. 2001), are one of the most economically important groups of teleosts facing heavy pressure from both commercial and recreational fisheries globally. Physiological and hydrodynamic adaptations (Magnuson 1973; Videler 1993; Dickson 1995; Donley et al. 2004; Pavlov et al. 2017; Gleiss et al. 2019), regional endothermy (Block et al. 1993), and a cold‐adapted cardiovascular system (Galli et al. 2009; Shiels et al. 2011) have enabled these large pelagic teleosts to inhabit broad geographical ranges and reign as oceanic apex predators from the surface to the mesopelagic (twilight) zone (Brill et al. 2005; Evans et al. 2008; Bernal et al. 2017). However, little is known about how tunas (*Thunnus* spp.) use their sensory systems to guide their behavior and navigate their three‐dimensional open ocean habitat, despite their economic and ecological significance. Previous studies of tunas have concentrated on the visual system due to their large eyes (Tamura and Wisby, 1963; Kawamura et al. 1981; Kröger et al. 2009) and well‐developed optic tectum (Kawamura et al. 1981; Lisney and Collin, 2006; Ha et al. 2026). However, light is strongly absorbed and scattered within the water column, which significantly limits visibility of prey, predators, and conspecifics even in clear, well‐lit oceanic water (Lythgoe, 1988; Warrant and Locket, 2004; Nilsson et al. 2014). Atema (2003) suggests that successfully navigating this open environment requires the integration of different senses and that the search for prey is mediated by the detection of a series of prey signals, including visual, chemical, and acoustic cues.

Underwater sound is generated by the movement or vibration of any immersed object, which imparts kinetic energy to the surrounding medium (Hawkins 1986, 2023). This local mechanical disturbance propagates as an acoustic wave, which alternately forces the component particles of the medium together and then apart (i.e., sound particle motion) (Hawkins 1986, 2023). The to‐and‐fro motion of component particles creates regions of compression and rarefaction, which leads to pressure fluctuations within the medium (i.e., sound pressure) (Hawkins 1986, 2023). Therefore, sound consists of two inseparable but distinct components: sound particle motion and sound pressure. Particle motion, which has both magnitude and direction, represents the vector (or directional) component of sound, while sound pressure, acting in all directions, represents the scalar component (Hawkins, 2023). The dualistic nature of sound is critical in fish audition as each is perceived via different anatomical pathways by fishes (Schellart and Popper, 1992; Sand and Bleckmann, 2008; Hawkins, 2023).

Particle motion is directly detected by the otolithic end organs in the inner ears (Schellart and Popper, 1992). Inner ears of fishes typically consist of three semicircular canals and three otolithic end organs (saccule, lagena, and utricle), where the former detect the vestibular signals, while the latter detect the acoustic signals (Popper et al. 2003). Each otolithic end organ is a membranous pouch consisting of a dense calcareous otolith, a sensory epithelium (macula) covered with large numbers of mechanoreceptive hair cells, and an otolithic membrane connecting the two (Popper 1977, 1981; Schellart and Popper, 1992; Hawkins and Popper, 2020). Apart from the otolith, the density of a fish's soft tissue is the same as the surrounding medium. Therefore, soft tissues including the macula move back and forth with the particle motion of a sound wave (i.e., acoustically transparent), while the otolith lags behind. The relative motion between the otolith and the underlying macula bends the ciliary bundle of the hair cells, and fishes detect sound particle motion as these mechanical stimuli are transmitted to the brain via the octaval nerve (cranial nerve VIII) (Popper et al. 1988; Schellart and Popper, 1992; Rogers and Zeddies, 2008). As all fishes have inner ears, particle motion can be detected by all fishes and is considered as the primary component of sound “heard” by fishes (Ladich, 2014; Popper and Hawkins, 2018).

Each ciliary bundle of hair cells has numerous stereocilia, arranged in rows of increasing height, and one kinocilium, placed at the extremity of the bundle (Popper, 1977; Popper and Fay, 2011). This defines the orientation of a hair cell, as the deflection of stereocilia toward the kinocilium (*θ* = 0°) causes a pronounced depolarization, while the deflection in the opposite direction (*θ* = 180°) causes a pronounced hyperpolarization (Hawkins and Popper, 2018). A minimum response is achieved when the deflection is orthogonal to its orientation (*θ* = 90°, 270°) (Hawkins and Popper, 2018). Each mechanoreceptive hair cell bundle is, therefore, a cosine vector detector, as the response from each hair cell is proportional to the cosine of the angle between the direction of propagating sound particle motion and the hair cell orientation (Rogers and Zeddies, 2008). Hair cells of the same orientation are organized together in a particular region of the macula, creating diverse hair cell orientation patterns, primarily in the saccule in teleosts (Popper and Coombs, 1982; Popper et al. 1988, 2003). As a result, each direction of incident particle motion elicits a distinct spatial pattern of receptor activation across the macula, and by weighting vectorial input from different regions of the maculae, fishes are thought to compute the direction of the particle motion of the incident sound (Sand and Bleckmann, 2008; Hawkins and Popper, 2018; Popper and Hawkins, 2018). The vector weighting model of directional hearing, however, has one critical challenge: the 180° ambiguity. Due to the to‐and‐fro motion of the particle motion, hair cells are not only deflected in the direction of the propagating sound wave, but also in the opposite direction (Popper et al. 1988; Rogers and Zeddies, 2008; Popper and Hawkins, 2018). Therefore, while the otolithic end organs can accurately capture the axis of sound propagation, they cannot resolve which direction along the axis the sound is travelling. A variety of hypotheses have been proposed as solutions to the 180° ambiguity (Schellart and de Munck, 1987; Kalmijn, 1988; Rogers and Zeddies, 2008), one of which involves the detection of sound pressure via the swimbladder (Schuijf and Buwalda, 1975; Veith et al. 2024).

Detecting sound pressure underwater demands two anatomical requirements: a gas‐filled chamber (e.g., swimbladder) and a close connection between the gas‐filled chamber and the inner ears (Schellart and Popper, 1992; Ladich, 2014; Hawkins, 2023). The gas in the swimbladder is compressed and rarefied by the incident sound pressure wave, and the vibration of the swimbladder is transmitted to the otolithic end organs either via an accessory structure such as the Weberian ossicles or a rostral projection of the swimbladder toward the inner ears (Schellart and Popper, 1992; Ladich, 2014). The transmitted vibration creates relative motion between the otolith and the underlying hair cells, triggering the nervous system. Therefore, sound pressure is detected in inner ears via an indirect pathway, translated into particle motion via the swimbladder (Popper et al. 1988). Comparing the direct stimulation induced by sound particle motion and indirect stimulation induced by sound pressure of the otolithic end organs allows fish to discern the direction of sound propagation and resolve the 180° ambiguity (Schuijf and Buwalda, 1975; Veith et al. 2024). In addition, the ability to detect sound pressure markedly enhances auditory sensitivity and extends the effective hearing range (Schulz‐Mirbach et al. 2012; Ladich and Fay, 2013). Swimbladder‐inner ear coupling has evolved independently, and with varying morphologies, among several teleost taxa (Ladich and Schulz‐Mirbach, 2016). These species, often associated with the advanced hearing abilities, have collectively been referred to as “hearing specialists” in contrast to “hearing generalists” that either lack a swimbladder or have a swimbladder that is not physically coupled to the inner ear (Schellart and Popper, 1992; Braun and Grande, 2008).

Tunas, apart from species like *Katsuwonus pelamis* (skipjack tuna) and *Euthynnus affinis* (mackerel tuna), possess a swimbladder, where its shape and size vary among species (Gibbs and Collette, 1967; Magnuson, 1973). However, no direct connection or even a close proximity between the swimbladder and the inner ears has been reported in these taxa. The inner ear morphology has been investigated in only two tuna species, *K. pelamis* (Popper, 1981) and Atlantic bluefin tuna, *Thunnus thynnus* (Song et al. 2006). In the saccule, the largest otolithic end organ, hair cells are organized in a “standard” hair cell orientation pattern in both species. A “standard” pattern, which is the most common pattern among teleosts (Schellart and Popper, 1992), has hair cells organized into four quadrants, where two horizontal groups are situated at the rostral part of the macula, while two vertical groups occur at the caudal part (Popper and Coombs, 1982; Popper et al. 2003). A “standard” pattern is often associated with the “hearing generalists” as it is found in species that lack accessory hearing structures (Schellart and Popper, 1992; Popper et al. 2003). The “hearing specialists” typically have “opposing” or “vertical” patterns (see Popper and Coombs, 1982). Behavioral measurements of the hearing thresholds of *Thunnus orientalis* (Pacific bluefin tuna), *Thunnus albacares* (yellowfin tuna) and *E. affinis* show that the frequency of greatest sensitivity (*f_b_*) is from 400 to 500 Hz (Iversen, 1967, 1969; Dale et al. 2015), which falls within the higher end of the spectrum for hearing generalists (Hawkins and Johnstone, 1978; Jerkø et al. 1989; Schulz‐Mirbach and Ladich 2013). The hearing range of tunas also does not exceed far beyond 1 kHz (Iversen, 1967; Dale et al. 2015). Hence, drawing from morphological and behavioral evidence, tunas are likely to be “hearing generalists” (Popper, 1981; Song et al. 2006). This, however, does not mean that tunas are oblivious to ambient noise, including anthropogenic noise, as *T. thynnus* display erratic behaviors, such as increased swimming speed, quick turns, and changes in depth, when exposed to various anthropogenic sounds (Sarà et al. 2007; Puig‐Pons et al. 2021).

Sound has been suggested as a more appropriate sensory signal for underwater communication than other signals, thanks to the minimal loss of information via absorption (only 1 dB of attenuation in 100 km of propagation in seawater), minimal impedance by physical barriers, and fast transduction velocity (Tavolga, 1971; Rogers and Cox, 1988; Hawkins, 2023). Being a vector (directional) signal, the ability to detect the direction of sound, especially in the far‐field, would be particularly beneficial for tunas to navigate the open ocean. It is, however, difficult to understand the functional significance of sound to tunas, as very little is known regarding what tunas are listening to. Morphologically, the inner ears of tunas suggest that they are likely “hearing generalists,” but only two species (*K. pelamis* and *T. thynnus*) have been investigated (Popper, 1981; Song et al. 2006), and the density of hair cells has been quantified only in *T. thynnus* (Song et al. 2006). Therefore, to better understand the significance of hearing in tunas, it is important to investigate the inner ears of other species to find a connection between structure and function, especially when it is extremely difficult to calibrate and perform behavioral or electrophysiological experiments on live animals (Dale et al. 2015). In addition to the orientation pattern of hair cells, or hair cell densities in maculae, morphological features of the inner ears such as the shape and size of the otoliths and the relative coverage of the maculae by the otoliths should also be investigated as they are hypothesized to enhance hearing ability and even resolve the 180° ambiguity without involving the swimbladder (Schellart and de Munck, 1987; Rogers and Zeddies, 2008). Hence, this study aims to (1) qualitatively and quantitatively investigate the inner ear (gross morphology; macular anatomy; and the abundance, distribution, characterization, orientation, and innervation of hair cells) in southern bluefin tuna, *Thunnus maccoyii*, and (2) discuss these results with respect to adaptations to hearing within a pelagic environment.

## 2 Methods

### 2.1 Source of Animals and the Preservation and Removal of the Inner Ear

Six juvenile individuals (92–100 cm in fork length, *L*_F_, and 15–20 kg in weight) and three adult individuals (152–190 cm *L*_F_ and 70–122 kg in weight) of *T. maccoyii* were caught from Bass Strait, Victoria, Australia, by recreational fishermen, and the heads donated under Victorian Fisheries Authority Research Permit #1447. The heads of all juvenile specimens were immersed directly into chilled fixative (either 4% paraformaldehyde in 0.1 M phosphate buffer [PFA] or modified Karnovsky's [2% paraformaldehyde and 2.5% glutaraldehyde in 0.1 M phosphate buffer]). The heads of the adult specimens, due to their large size, were kept on ice and transferred to the laboratory at La Trobe University (Melbourne) as soon as possible (typically within 12–24 h). Upon arrival, the inner ears were immediately dissected from the large heads and stored in 4% PFA. Inner ears from all nine specimens were used to conduct the gross anatomical study, and the left and right ears from the two adult specimens (152 and 176 cm *L*_F_) were further analyzed using scanning electron microscopy (SEM).

A portable electric saw (Bosch 12 V cordless NanoBlade Saw) and a handsaw were used to remove the lower jaw and the rostral part of the head in front of the eyes so that the head remained upright and stable during the dissections. Boning knives and large rongeurs were used to remove the soft tissue surrounding the cranium and to open the roof of the neurocranium to expose the semicircular canal portion of the inner ears sitting lateral to the cerebellum. Finer rongeurs, microforceps, and iridectomy scissors were then used to carefully remove the adipose tissue and ossified otic capsules tightly surrounding all three semicircular canals and end organs. The inner ears were removed from the cranial cavity by severing the different branches of the auditory nerve (nVIII) and placed into cold fixative (4% PFA in 0.1 M phosphate buffer) for 48–72 h. Using a pipette, fixative was frequently drizzled directly onto the exposed brain and inner ears throughout the long dissection process in order to preserve the quality of the tissues.

To record the gross anatomy and make morphometric measurements, the inner ears of *T. maccoyii* were photographed using a Nikon SMZ 745T stereomicroscope with fiber optic illumination, while immersed in 0.1 M phosphate buffer. Morphometric measurements of the semicircular canals, otoliths, and otolithic membranes were analyzed using ImageJ (64‐bit Java, version 2.16.0/1.54). The canal radius and bore radius were measured at the midpoint of the horizontal, anterior, and posterior semicircular canals. The otoliths were photographed with their otolith membranes still attached to calculate the relative area of the otolithic membrane covered by the otolith.

### 2.2 SEM

Prior to SEM, the fixed maculae of two adult specimens (SBT19 and SBT20) were rinsed in three changes of 0.1 M Sorensen's buffer (30 min each) and post‐fixed in 1% osmium tetroxide (OsO_4_) in 0.1 M Sorensen's buffer for 2 h. After post‐fixation, the maculae were rinsed in two changes of Milli‐Q water (30 min each) and dehydrated through a graded series of ethanols (30%, 50%, 70%, 80%, 90%, 95%, and three changes of 100% ethanol). Maculae were dehydrated for 30 min for each step, except for 70%, when the tissues were dehydrated overnight. A platform rocker was used throughout the dehydration process. The maculae were then critical point dried in a Quorum E3000 critical point drier using liquid CO_2_ as the intermediary fluid. Dried maculae were mounted onto aluminum stubs, sputter‐coated with a 22 nm layer of platinum using a SC7640 sputter coater, and imaged using a Hitachi SU7000 FE SEM.

### 2.3 Mapping and Quantification of Macular Hair Cell Bundles

To capture the shape and calculate the surface area of the saccule, lagena, and utricle, a montage of low magnification images (x50–60) was taken, as the lowest magnification available (x50) could not capture the entire surface of any macula. The montage of each macula was then “stitched” together in Adobe Illustrator (version 29.5), referring to the markers that were placed during the low magnification imaging. The areas of the saccule, lagena, and utricle were calculated by tracing the outline of maculae using the polygon selection tool in ImageJ (64‐bit Java, version 2.16.0/1.54) (RRID: SCR_003070).

A series of high magnification (2K–4K) images with corresponding areas of 3,024‐758 µm^2^, respectively, were taken along transects that were perpendicular to the long axis of the saccule and lagena. For the left and right saccule of SBT19, four to seven sites were selected for imaging along each of 16 transects, depending on the length of the transects and the quality of the tissue within each transect. For the left and right saccule of SBT20, three to eight sites were selected for imaging along each of 21 transects. For the lagena and utricle, the quality of preservation was not good enough to quantify the number of hair cells in SBT19, and, therefore, only the surface areas were calculated. For the left and right lagena of SBT20, five to seven sites were selected for imaging along each of nine transects. For the left and right utricles of SBT20, which is shaped like a dented apostrophe, images were taken in a grid‐like fashion, where each sampled location was equally spaced. In total, 95 and 100 sites were sampled in the left and right utricles, respectively. For every sampled site in each macula, hair cell number and density, hair cell type, and the orientation of hair cell bundles were all recorded.

To visualize the distribution pattern of hair cell density and type, iso‐density contour maps were created using a custom‐written script in Python (Python Language Reference, version 3.12.4, written by Lucille Chapuis using scipy, matplotlib, and read_roi modules). The outline of each macula was obtained using the polygon‐selection tool in ImageJ (64‐bit Java, version 2.16.0/1.54), and the *x* and *y* coordinates of each sampled site were obtained using the multipoint tool. Hair cell density (total number of hair cells in the area of 3024 µm^2^) and type were then assigned to each corresponding coordinate, forming the input dataset. Hair cell types were determined based on kinocilium and stereocilia length, following the classification criteria of Popper (1977). The radial basis function (RBF) interpolator algorithm with the default thin plate spline kernel was used to both interpolate and extrapolate the input data over the whole macula. Contour maps were generated for each hair cell type and the combined hair cell types. The hair cell orientation maps were also generated by manually placing the arrows corresponding to the hair cell orientation at each site using Adobe Illustrator (version 29.5).

As the density of ciliary bundles vary within each macula, it was crucial to identify the regions of high and low densities to avoid over or under estimation of the total number of hair cells within each macula (Popper and Hoxter, 1984). To quantify the total hair cell number, each macula was first divided into high‐ and low‐density areas, which were determined from the low magnification images and the iso‐density contour maps. Then, each macula was further subdivided based on its hair cell orientation groups. This divided the saccule into six subareas (High_Horizontal, Low_Horizontal, High_Vertical, Low_Vertical, High_Diagonal, and Low_Diagonal) and lagena and utricle into four subareas (High_Dorsal, Low_Dorsal, High_Ventral, and Low_Ventral and High_Lateral, Low_Lateral, High_Medial, and Low_Medial, respectively). Hair cell number (*H*) in each subarea was then estimated by using the extrapolation equation used in Popper and Hoxter (1984)

$$H=\sum C_{h}\;\left(A_{h}/\Sigma P_{h}\right)+\sum C_{l}\;\left(A_{l}/\Sigma P_{l}\right)$$

where C*_h_* and C*_l_* are the number of cells in each high‐density and low‐density micrographed region, respectively; P*_h_* and P*_l_* are the areas of each of the micrographed regions; and A*_h_* and A*_l_* are the total high‐density and low‐density areas for the two regions.

The total number of hair cells for each macula was calculated by summing hair cell numbers extrapolated from each subarea. Hair cell number and surface area for each orientation group and low density versus high density were also calculated.

### 2.4 Magnetic Resonance Imaging (MRI)

To check the in situ morphology and orientation of the saccule and lagena within the neurocranium, the heads of two additional small tunas (88 and 95 cm *L_F_*) were imaged using MRI. In preparation for MRI, the PFA‐fixed *T. maccoyii* heads were immersed in 10 L of phosphate‐buffered (PB) azide solution (0.1 M, 0.01%) for 3 weeks to leach out the fixative. Then, 8 L of new PB azide solution (0.1 M, 0.01%) was made, and 43.76 g of diethylenetriaminepentaacetic acid gadolinium (III) dihydrogen salt was added to create a PB azide solution with 10 mM of gadolinium salt. The heads were then immersed in the solution for 8 weeks to stain the ear and brain tissue inside the cranium. Eyes and snout were removed to reduce the volume of the head and to facilitate the diffusion of staining agents. The MRI data were acquired on a Siemens (Germany) Magnetom 7T Plus system, in a 1Tx/32Rx Head coil (Nova Medical) using a turbo FLASH sequence (Haase et al. 1986) at The University of Melbourne. The 3D MRI data consisted of 0.2 mm isotropic voxels with a 200 mm × 200 mm in‐plane field of view and 192 slices (100% slice oversampling) acquired with 7/8 partial Fourier coverage in the slice and phase encode directions. The repetition time was 13 ms with an echo time of 5 ms. To improve signal‐to‐noise, four averages were acquired, resulting in a total imaging time of 2 h and 14 min. The images were then analyzed using Dragonfly (Comet Technologies Canada Inc., version 2024).

## 3 Results

### 3.1 Gross Morphology of the Inner Ear

The inner ears of *T. maccoyii* (Figure 1a) are adjacent and positioned laterally to the brain (Figure 1b). The inner ears fill a large portion of the volume of the cranial cavity, and the lateral view of the ears show that the size of the ears is comparable relative to the brain (Figure 1c). Three semicircular canals are positioned orthogonally to one another and connected to their respective ampullae (Figure 1c,d). The most superior end organ, the utricle, is connected to the anterior and horizontal ampullae, all of which form the upper inner ear (i.e., pars superior). The lower inner ear (i.e., pars inferior) comprises the saccule and the lagena, which are connected rostro‐caudally, and sit ventrolateral to the hindbrain (Figure 1c). The upper and lower ears are joined together at the narrow base of the common crus.

**FIGURE 1:**
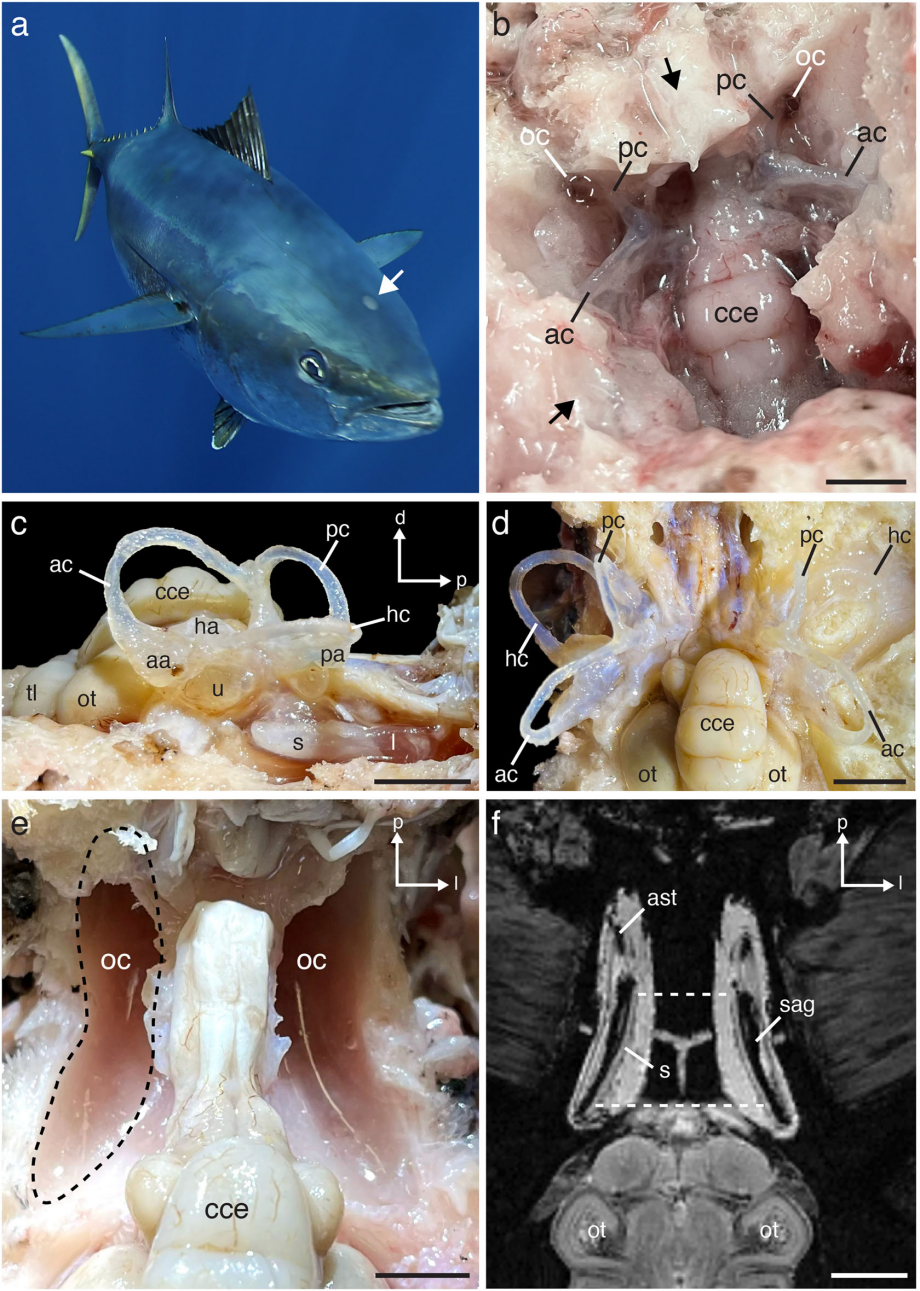
(a) Photograph of the southern bluefin tuna *Thunnus maccoyii* (provided, with permission, by Al McGlashan). Approximate position of the brain and inner ears (arrow), which is underneath the distinct pineal window situated on the dorsal surface of the head. (b) Photograph of partially exposed corpus cerebelli (cce) and semicircular canals of left and right inner ears. The roof of the neurocranium and thick layers of fatty tissues (arrows) were removed to expose the dorsal region of the inner ear. Note the otic capsules (oc) forming tunnels (dotted circle) tightly surrounding the semicircular canals. (c) Photograph showing the relative position of the brain and the inner ear (left) in lateral view. (d) Photograph showing the relative position of the brain and both inner ears in dorsal view. The right ear is fully exposed, while only the semicircular canals are exposed for the left ear. (e) Photograph of the otic capsules housing the lower inner ears (saccule and lagena). The outline of the end organ chambers (dotted line) shows that the rostral part is angled away from the midline. (f) Magnetic resonance image of the lower inner ears in transverse section. The distance between the left and the right saccules (dotted line) increases anteriorly. Orientation: d, dorsal; l, lateral; p, posterior. aa, anterior ampulla; ac, anterior canal; ast, asteriscus; ha, horizontal ampulla; hc, horizontal canal; l, lagena; ot, optic tectum; pa, posterior ampulla; pc, posterior canal; s, saccule; sag, sagitta; tl, telencephalon; u, utricle. Scale bars, 5 mm (b–f).

The otic capsule or bony labyrinth, composed of well‐ossified and fatty tissue, wraps around the upper inner ear, leaving very little space in between the internal walls of the otic capsule and the membranous labyrinths (Figure 1b). The lower inner ear is tightly surrounded by hard ossified tissue, forming a distinct and compact chamber, which is deeply oriented toward the ventral and medial walls of the cranium (Figure 1c,e). The chamber is not completely parallel to the midsagittal plane of the fish. Instead, the rostral part of the chamber is angled away from the midline by about 35°, with the angle decreasing posteriorly, eventually becoming parallel to the midline (Figure 1e). As there is little to no space in between the internal walls of the bony chamber and the membranous walls of the lower inner ears, it appears that the rostral ends of the left and right saccules are directed away from one another. The MRI images in the horizontal plane show that the in situ orientation of the lower inner ear matches the orientation of the chamber. The rostral part of the saccule is angled away from the midline, while the caudal part of the saccule and lagena are parallel to the midline (Figure 1f). The distance between the left and right saccules, estimated to be immediately medial to the otolith (sagitta), almost doubles from caudal (6 mm) to rostral (11 mm) as the maculae are angled away from the midline (Figure 1f).

The semicircular canals of *T. maccoyii* are well‐developed in terms of both their length and the thickness of their membranous walls, and the canals of mature *T. maccoyii* are cartilaginous (Figure 2a,b). Regardless of the size of the fish, the horizontal canal is the longest, followed by the anterior and posterior canals. The radius of each canal is comparable, and both canal length and canal radius increase with increasing fork length (Table 1). The three otolithic end organs (saccule, lagena, and utricle) of *T. maccoyii* are thick membranous pouches enclosing calcareous otoliths: the sagitta, asteriscus, and lapillus, respectively. The saccule and lagena are oriented in the vertical plane, and the utricle is oriented in the horizontal plane (Figure 2a,b). Within the vertical plane, the saccule and the lagena are oriented rostro‐caudally. The saccule is the largest end organ, and its height tapers anteriorly. The bowl‐shaped utricle is the second largest end organ, and the crescent‐shaped lagena is the smallest end organ.

**FIGURE 2:**
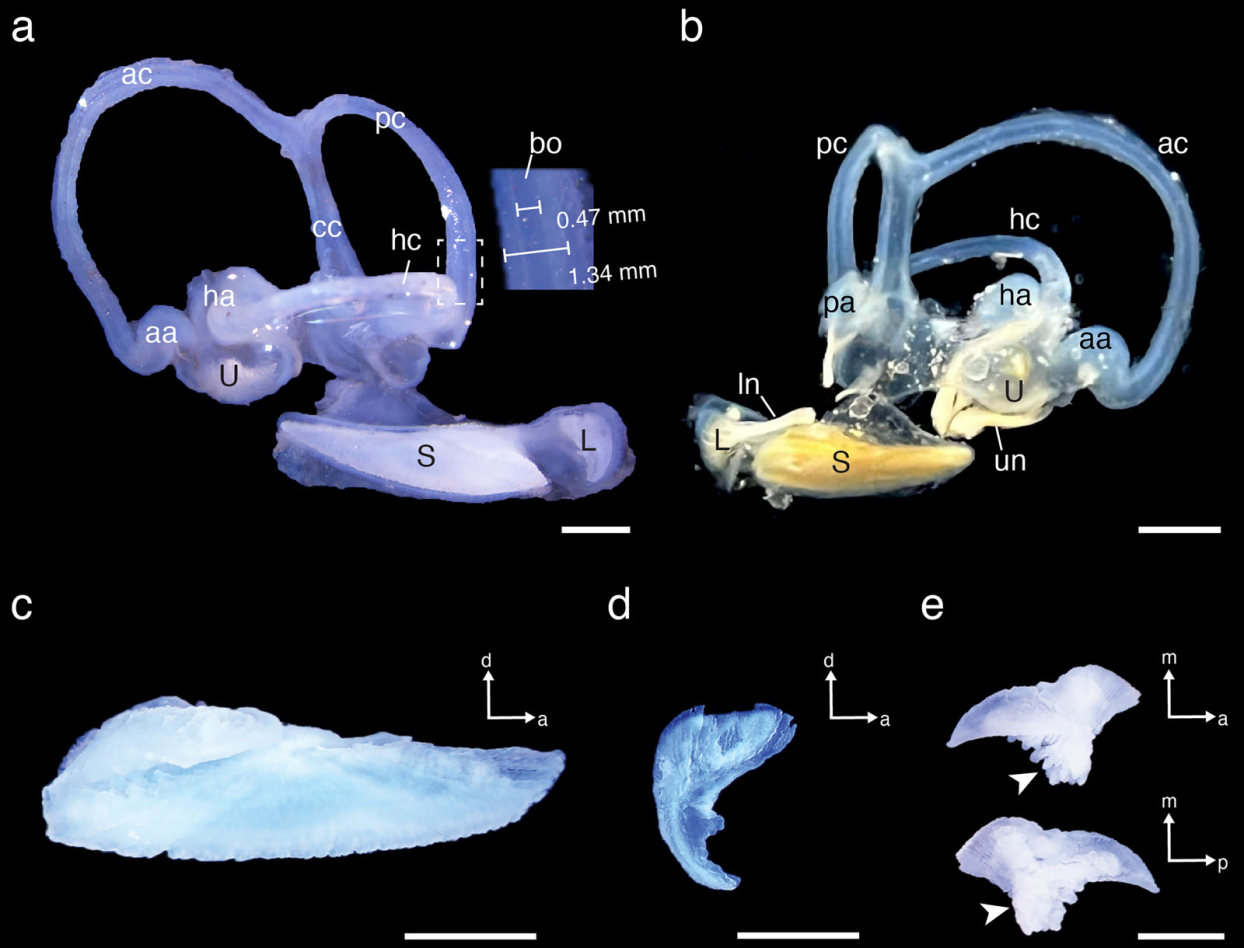
(a) Photograph of the left inner ear of a mature *Thunnus maccoyii* in lateral view. Portion of the posterior semicircular canal (dotted box) is magnified to display the bore (bo) and the thick walls of the semicircular canals. (b) Photograph of the left inner ear of a juvenile *T. maccoyii* in medial view. (c) Photograph of the sagitta in lateral view. (d) Photograph of the asteriscus in lateral view. (e) Photograph of the lapillus in dorsal (upper) and ventral (lower) views. Note the complex, bumpy ventral surface of the lapillus (arrowheads). Orientation: a, anterior; d, dorsal; m, medial; p, posterior. ln, lagenar nerve; un, utricular nerve. Scale bars, 5 mm (a, b); 4 mm (c); 3 mm (d); 2 mm (e).

**TABLE 1:** Information on size and semicircular canal dimensions of individual southern bluefin tuna (SBT), *Thunnus maccoyii*.

Fish ID	*L*_F_ (cm)	Weight (kg)	Side	Canal length (mm)			Canal diameter (mm)		
				Anterior	Horizontal	Posterior	Anterior	Horizontal	Posterior
SBT12	100	20	R	22	—	—	1.0	—	—
SBT13	94	15	L	18	23	18	0.9	0.9	0.9
SBT15	92	15	L	22	24	16	1.1	0.9	0.9
SBT16	92	15	L	22	26	17	1.0	0.9	0.9
SBT17	100	20	R	25	29	17	0.9	1.0	1.0
SBT18	95	15	L	23	26	16	0.8	0.9	0.8
**Juvenile SBT (mean ± SE)**	**95 ± 1**	**17 ± 1**		**22 ± 1**	**26 ± 1**	**17 ± 0**	**1.0 ± 0.0**	**0.9 ± 0.0**	**0.9 ± 0.0**
									
SBT19	152	70	R	27	32	20	1.2	1.2	1.3
SBT20	176	96	R	36	37	25	1.3	1.3	1.3
SBT21	190	122	R	33	38	28	1.5	1.4	1.4
**Mature SBT (mean ± SE)**	**173 ± 11**	**96 ± 15**		**32 ± 3**	**36 ± 2**	**24 ± 2**	**1.3 ± 0.1**	**1.3 ± 0.1**	**1.3 ± 0.0**

*Note:* Means and standard errors are in bold.

Abbreviations: L, left ear; *L*_F_, fork length; R, right ear.

### 3.2 Morphology of the Otoliths

The otolith in each end organ has its own distinctive shape. The sagitta is elongated, and its height tapers toward the anterior end (Figure 2c). The surface of the sagitta is covered with complex protuberances. The depth profile also changes along the longitudinal axis as the otolith increases in thickness posteriorly with a prominent protrusion at the upper lateral surface (Figure 2c). The asteriscus is crescent‐shaped and its width tapers toward the ventral end. Its surface also has a complex depth profile with a small protrusion at the anterior surface (Figure 2d). The lapillus is “ginkgo‐leaf” shaped, with its pointed end extending posteriorly (Figure 2e). The profile of its dorsal surface is relatively smooth, but its ventral surface, which is facing the sensory epithelium, is covered with distinctive bumps of varying heights, which are deepest at the petiole‐like region. The size of the sagitta grows as the size of the fish increases, and the sagittas of mature *T. maccoyii* are about twice as large as those of juvenile *T. maccoyii* (Table 2). The sagitta, regardless of the size of the fish, possesses a gelatinous material on its medial surface (Figure 3a). Considering its position between the otolith and the macula, it was suspected to be an otolithic membrane. A high‐magnification SEM image showed that this jelly‐like material has a porous mesh‐like composition, and hair cells are found within the pores, corroborating its identity as an otolithic membrane (Figure 3b).

**TABLE 2:** Quantitative assessment of the area of the sulcus (S) and otolith (O) of juvenile and mature *T. maccoyii*.

Fish ID	Sagitta		
	**Otolith (mm^2^)**	**Sulcus (mm^2^)**	**S:O ratio**
SBT12	32.4	15.6	0.5
SBT13	33.5	16.1	0.5
SBT18	34.7	15.2	0.4
**Juvenile SBT (mean ± SE)**	**33.5 ± 0.7**	**15.6 ± 0.3**	**0.5 ± 0.0**
			
SBT19	60.5	26.2	0.4
SBT20	72.9	31.8	0.4
SBT21	80.5	34.8	0.4
**Mature SBT (mean ± SE)**	**71.3 ± 5.8**	**30.9 ± 2.5**	**0.4 ± 0.0**

*Note:* All sagittas were collected from the right ear. Means and standard errors are in bold.

**FIGURE 3:**
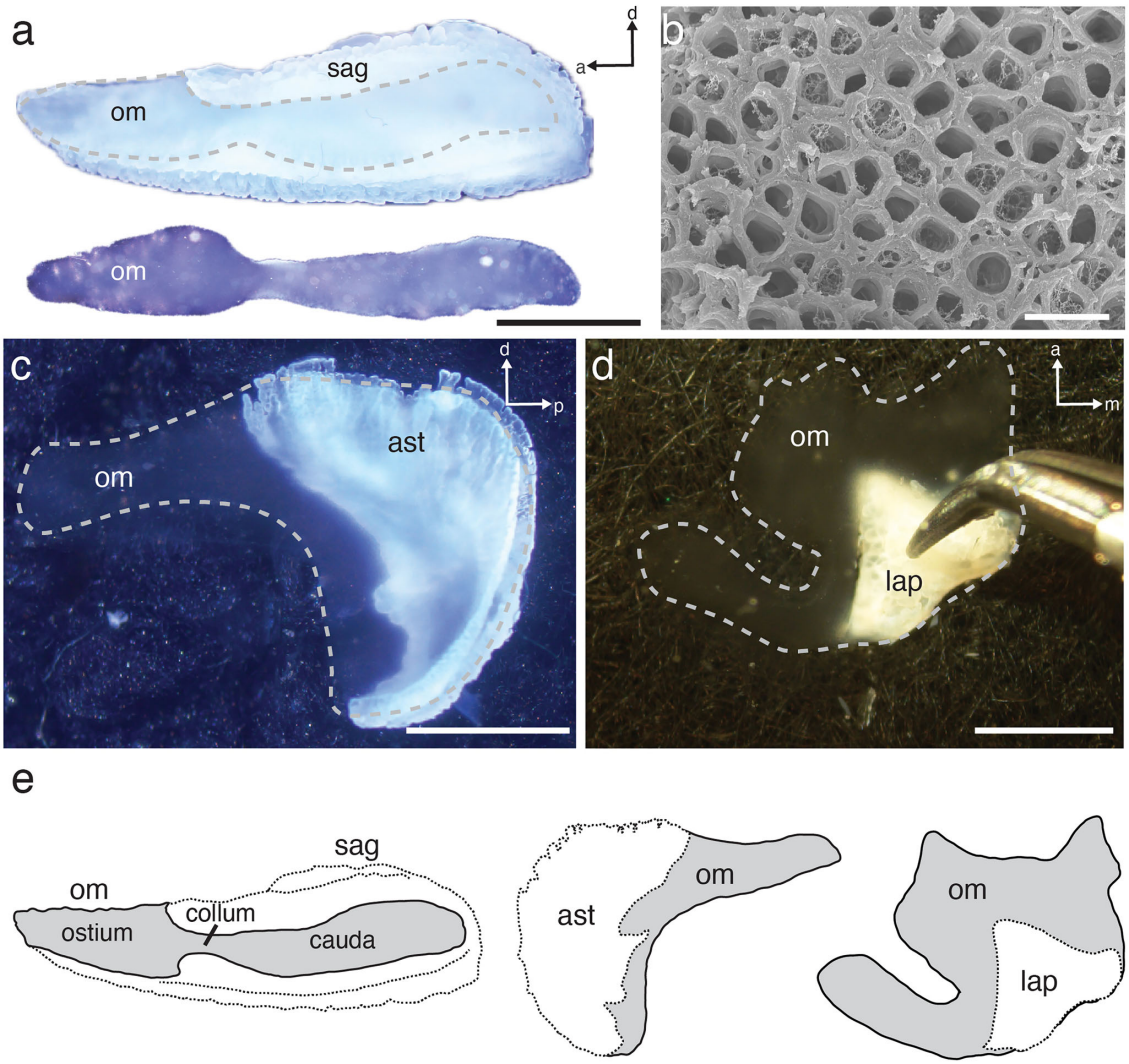
(a) Photograph of the sagitta in medial view (up) and the dissected otolithic membrane (om). Note the translucent otolithic membrane (outlined) completely fits into the sulcus. (b) Scanning electron micrograph of the otolithic membrane surface showing the porous mesh‐like surface. (c) Photograph of the asteriscus in medial view. Note that the otolithic membrane (outlined) extends beyond the overlying otolith anteriorly. (d) Photograph of the lapillus in dorsal view. The otolithic membrane also extends beyond the overlying otolith. (e) Schematic drawings (not to scale) illustrating the variation in coverage of the otolithic membranes by the otoliths. The sagitta completely covers the otolithic membrane, which fills up the sulcus, while the asteriscus and the lapillus only partially cover the otolithic membranes. Morphological structures of the sulcus (ostium, collum, and cauda) are labeled. Orientation: a, anterior; d, dorsal; m, medial; p, posterior. ast, asteriscus; lap, lapillus; sag, sagitta. Scale bars, 4 mm (a); 5 µm (b); 2 mm (c); 2.5 mm (d).

Removal of the otolithic membrane of the saccule, which perfectly fits into the sulcus area (i.e., sulcus acusticus) of the sagitta, allowed the characterization of the sulcus structure. The sulcus comprises about 45% (*n* = 6) of the medial surface of the sagitta (mean sulcus (S) to otolith (O) ratio S:O of 0.45; Table 2). The sulcus opening type is ostial, where the ostium is open toward the anterior margin, and the sulcus margin type is archaesulcoid, as the ostium and cauda have a similar height and there is no clear division between them (Schwarzhans, 1978; Schwarzhans et al. 2017). The otolithic membranes are also attached to the medial surface of the asteriscus and the ventral surface of the lapillus (Figure 3c,d). Interestingly, unlike the sagitta, the asteriscus and lapillus only cover a portion of their respective membranes. Most of the anterior one‐third of the lagena's otolithic membrane is not covered by the asteriscus, which comprises about 30% of the total membrane area. The lapillus covers even less area of the membrane, since about 70% of the otolithic membrane of the utricle is uncovered (Figure 3e; Table 3).

**TABLE 3:** Quantitative assessment of the area of otolithic membrane (OM) covered by the otoliths (O) (i.e., asteriscus and lapillus) in three mature *T. maccoyii* specimens.

Fish ID	Side	Asteriscus			Lapillus		
		**Otolithic membrane (mm^2^)**	**Otolith coverage (mm^2^)**	**O:OM ratio**	**Otolithic membrane (mm^2^)**	**Otolith coverage (mm^2^)**	**O:OM ratio**
SBT19	R	13.4	8.1	0.6	20.0	7.4	0.4
SBT20	R	16.7	12.2	0.7	21.4	6.2	0.3
SBT21	L	14.0	11.6	0.8	23.0	5.9	0.3
	R	—	—	—	23.1	6.1	0.3
**Mean ± SE**		**14.7 ± 1.0**	**10.6 ± 1.3**	**0.7 ± 0.1**	**21.9 ± 0.7**	**6.4 ± 0.3**	**0.3 ± 0.0**

### 3.3 Sensory Epithelium and Hair Cell Orientation

The saccular macula is elongated and is the largest of the three epithelia with a mean area of 10.9 ± 0.8 mm^2^ (mean ± SE, *n* = 4) (Figure 4a; Table 4). The ciliary bundles form a rostro‐caudal (horizontal) axis in the arrowhead‐shaped rostral part of the macula, where the bundles are oriented caudally in the dorsal half and rostrally in the ventral half. The ciliary bundles form a rostrodorsal‐caudoventral (diagonal) axis in the elongated caudal part of the macula, where the bundles are oriented rostro‐dorsally in the dorsal half and caudo‐ventrally in the ventral half (Figure 4b,c). Each rostral and caudal part of the macula fits into the ostium and cauda of the sulcus, respectively, and the shape of the macula is identical to the shape of the overlying otolithic membrane (Figure 3a,e). An additional opposing group of hair cells is found within the relatively narrow intermediate region that connects the rostral and caudal parts of the macula, corresponding to the collum of the sulcus (Figure 4b,c). The ciliary bundles form a dorsoventral (vertical) axis in this “bottleneck”‐shaped intermediate region of the macula, where the bundles are oriented dorsally in the dorsal half and ventrally in the ventral half. The horizontal and diagonal hair cell groups dominate the saccular macula in terms of both area and number: the horizontal group occupies about 41% of the total area and hair cell number, while the diagonal group occupies about 54% of the total area and hair cell number. The vertical hair cell groups occupy about 5% of both the total area and total hair cell number (Table 4). To corroborate the innervation of the additional orientation group of hair cells, the VIIIth nerve bundles innervating the saccular macula were investigated. Previous inner ear morphology studies have discovered that each group of hair cells with opposing hair cell orientation in the saccular macula is innervated by a separate ramus of the VIIIth nerve (Saidel and Popper, 1983; Deng et al. 2013). Here, three separate nerve branches innervate the medial surface of the saccule, each corresponding to the three regions occupied by the opposing hair cell groups (Figure 5). Therefore, the saccular epithelium of *T. maccoyii* has ciliary bundles oriented in six directions (i.e., in three opposing groups) dividing the macula into six distinct compartments.

**FIGURE 4:**
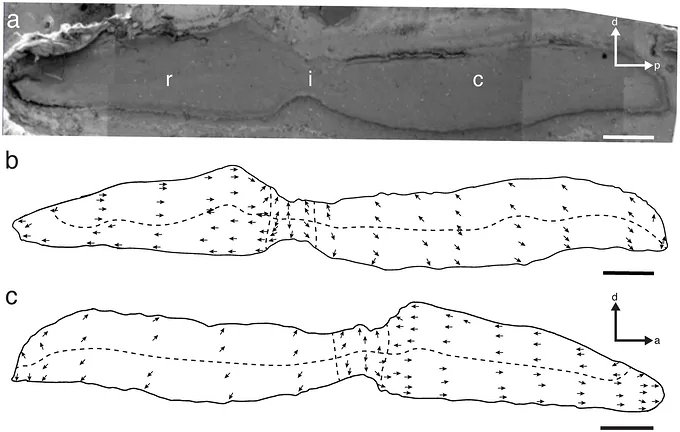
(a) Montage of low magnification scanning electron micrographs of the left saccular macula of *Thunnus maccoyii* (SBT20). The elongated macula can be separated into three parts (anterior, intermediate, and posterior) based on the hair cell orientation groups. (b) Hair cell orientation map of the left saccular macula of SBT20. (c) Hair cell orientation map of the right saccular macula of SBT20. Note that the hair cell orientation of the left and the right saccular maculae is symmetrical. The tip of the arrows indicates the direction of the kinocilium in hair cell bundles in that region. Orientation: a, anterior; d, dorsal; p, posterior. c, caudal; i, intermediate; r, rostral. Scale bars 892 µm (a, b); 880 µm (c).

**TABLE 4:** **|** Quantitative assessment of the surface area and hair cell numbers in saccule, lagena, and utricle of two mature *T. maccoyii* specimens. The macular regions are subdivided by their hair cell orientation groups.

		SBT19 (70 kg)		SBT20 (96 kg)		Mean ± SE
		L	R	L	R	
Saccule	Total area (mm^2^)	9.5	9.6	12.3	12.0	10.9 ± 0.8
	Horizontal orientation area	4.1	4.0	4.6	5.2	4.5 ± 0.3
	Diagonal orientation area	4.9	5.3	7.2	6.1	5.9 ± 0.5
	Vertical orientation area	0.5	0.3	0.6	0.7	0.5 ± 0.1
	Total hair cell number	195,176	198,051	247,803	242,367	220,000 ± 10,000
	Horizontal group number	82,354	78,642	94,787	108,047	91,000 ± 7000
	Diagonal group number	102,724	114,068	138,990	117,292	118,000 ± 8000
	Vertical group number	10,098	5341	14,026	17,028	12,000 ± 3000
Lagena	Total area (mm^2^)	3.8	3.7	5.3	5.2	4.5 ± 0.4
	Dorsal group area	1.6	1.5	2.3	2.4	2.0 ± 0.2
	Ventral group area	2.2	2.2	3.0	2.8	2.5 ± 0.2
	Total hair cell number	—	—	205,084	209,283	207,000 ± 2000
	Dorsal group number	—	—	91,457	96,995	94,000 ± 3000
	Ventral group number	—	—	113,627	112,288	113,000 ± 700
Utricle	Total area (mm^2^)	6.3	6.7	8.8	8.6	7.6 ± 0.6
	Lateral group area	—	—	4.1	4.2	4.1 ± 0.1
	Medial group area	—	—	4.7	4.4	4.6 ± 0.2
	Total number	—	—	315,729	315,997	315,900 ± 100
	Lateral group number	—	—	173,333	172,336	172,800 ± 500
	Medial group number	—	—	142,396	143,661	143,000 ± 600

Abbreviations: L, left ear; R, right ear; SE, standard error.

**FIGURE 5:**
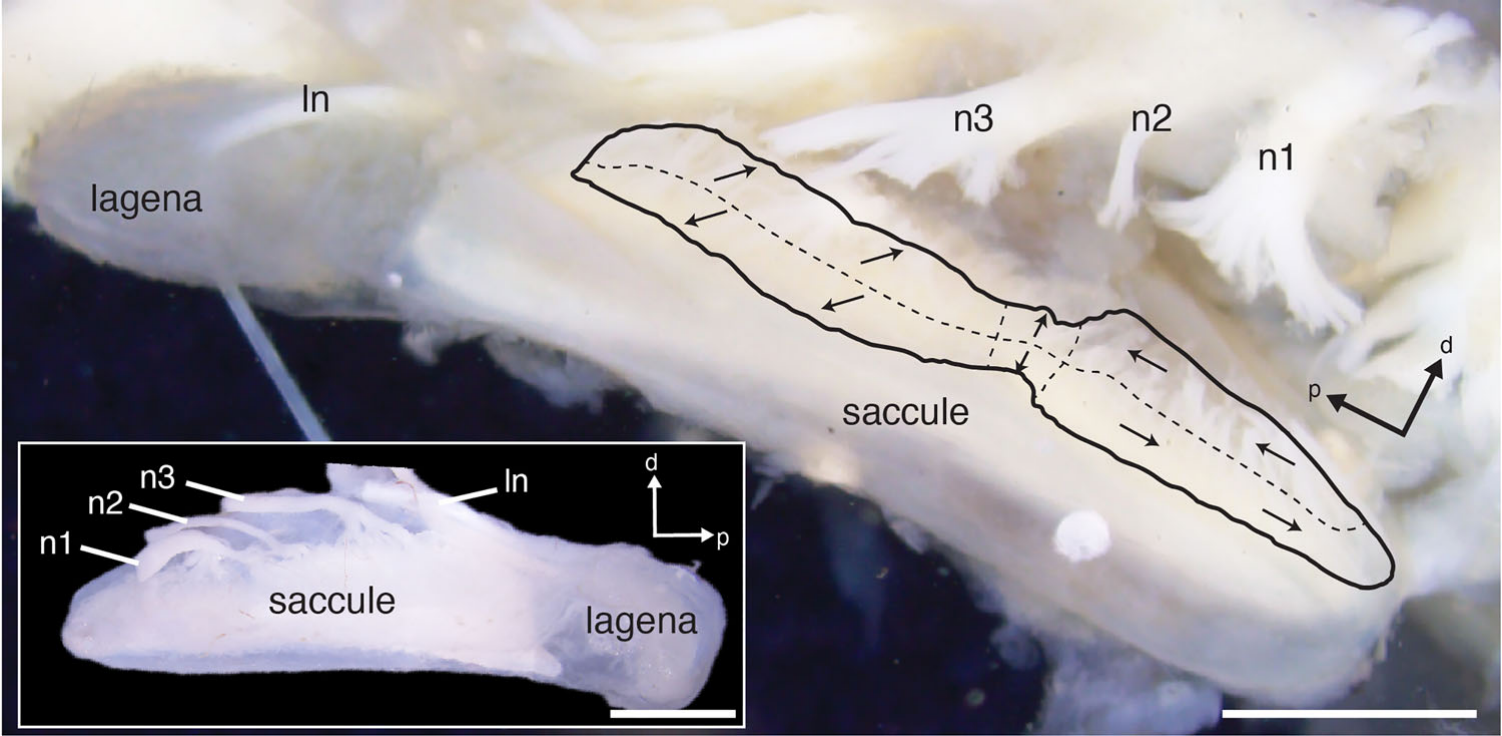
Photograph of the lower inner ear of a juvenile *Thunnus maccoyii* (SBT12) in medial view showing the innervation of the macular regions by the VIIIth cranial nerve. Three distinct nerve bundles (n1, n2, and n3) innervate the saccular macula, each of which corresponds to and emanates from a specific hair cell orientation group. Only one nerve bundle (ln) innervates the lagenar macula. Note that the nerve bundle innervating the intermediate part of the saccular macula (n2) is significantly smaller than the other nerve bundles (n1 and n3). Inset: photograph showing the lower inner ear of a mature *T. maccoyii* (SBT20) in lateral view, which also shows three distinct nerve bundles innervating the saccular macula and one bundle innervating the lagenar macula. Orientation: d, dorsal; p, posterior. Scale bars, 3 mm; 5 mm (inset).

The lagenar macula is crescent‐shaped and its anterior end faces the posterior region of the saccular macula (Figure 6a). It is the smallest of the three epithelia with a mean area of 4.5 ± 0.4 mm^2^ (*n* = 4) (Table 4). The lagena has one set of opposing hair cell groups. The ciliary bundles retain an orientation that is parallel to the closest edges of the epithelium so that, as the lagenar epithelium curves, the orientation of the bundles also shifts (Figure 6b,c). Thus, despite having only one opposing group of hair cells, the lagena technically has hair cells that are oriented in horizontal, vertical, and diagonal axes, similar to the saccule. The nominal dorsal hair cell group occupies about 46% of the total lagenar area and total hair cell number, and the nominal ventral hair cell group occupies about 54% of the total macular area and hair cell number (Table 4). When compared to the area of the lagenar otolithic membrane that is uncovered by the asteriscus, it is primarily horizontally oriented hair cells and a small portion of dorsally and rostro‐dorsally oriented hair cells that are uncovered by the otoliths (Figure 6d). Only one branch or ramus of the VIIIth nerve innervates the lagena, which is expected from the number of opposing groups of hair cells (Figure 5).

**FIGURE 6:**
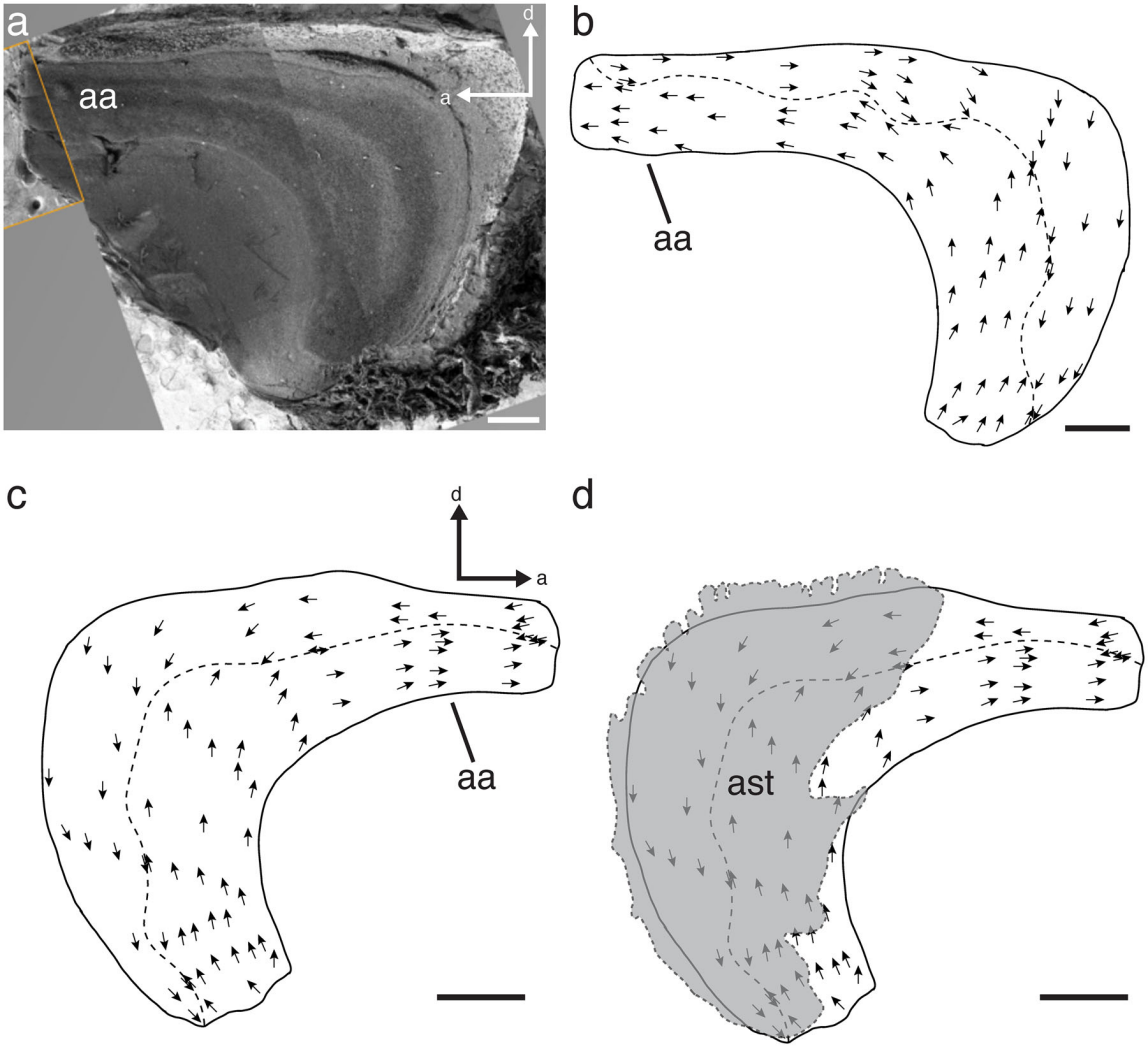
(a) Montage of low magnification scanning electron micrographs of the left lagenar macula of *Thunnus maccoyii* (SBT20). The crescent‐shaped macula has an extension toward the anterior (anterior arm). (b) Hair cell orientation map of the left lagenar macula of SBT20. (c) Hair cell orientation map of the right lagenar macula of SBT20. (d) Lagenar macula overlain by the otolith. Note that the horizontally oriented hair cells in the anterior arm are primarily uncovered. Orientation: a, anterior; d, dorsal. aa, anterior arm; ast, asteriscus. Scale bars, 520 µm (a, b); 616 µm (c, d).

The utricular macula is bowl‐shaped and has a tail‐like lacinia projecting laterally toward the horizontal ampullae (Figure 7a). The utricular macula has a mean area of 7.6 ± 0.6 mm^2^ (*n* = 4) (Table 4). In the utricle, two hair cell groups face each other, where the hair cells in the medial part of the macula face laterally, while the hair cells in the lateral part of the macula face medially (Figure 7b,c). The lateral hair cell group comprises about 48% of the total macular area and 55% of the total hair cell number, and the medial hair cell group comprises about 52% of the total macular area and 45% of the total hair cell number (Table 4). Similar to the lagena, despite having only one set of opposing hair cell groups, the hair cell bundles in the utricle cover the horizontal, vertical, and diagonal axes along the horizontal plane of the fish. When compared to the area of the utricular otolithic membrane that is covered by the lapillus, the lapillus primarily covers the medial group of hair cells that are facing the lateral direction (Figure 7d).

**FIGURE 7:**
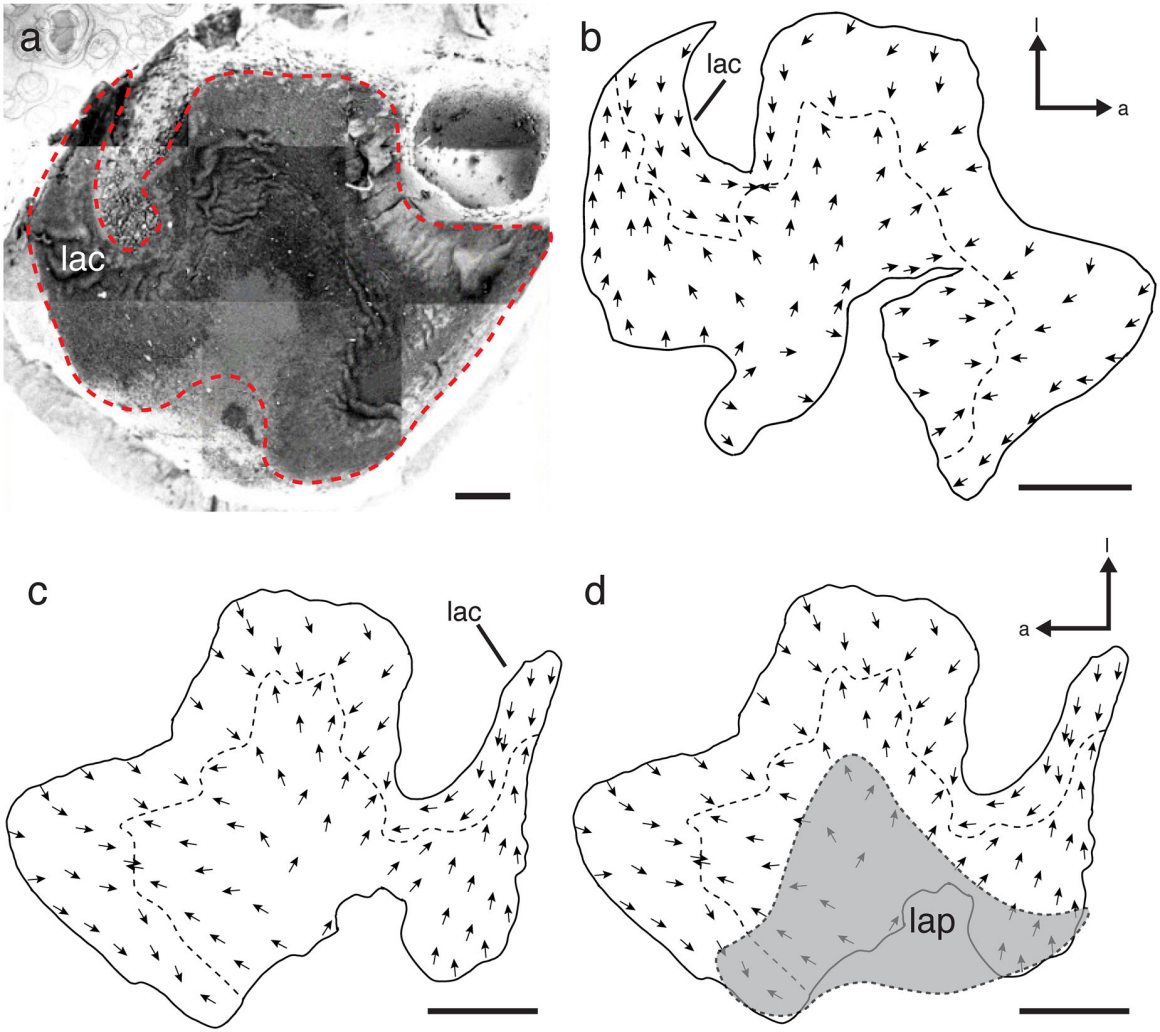
(a) Montage of low magnification scanning electron micrographs of the left utricular macula of *Thunnus maccoyii* (SBT20). The bowl‐shaped macula has a tail‐like extension (lacinia), laterally. (b) Hair cell orientation map of the left utricular macula of SBT20. The medial parts of the utricle were cut to flatten out the hemispherical macula for SEM analysis. (c) Hair cell orientation map of the right utricular macula of SBT20. (d) Utricular macula overlain by the otolith. Note that the hair cells in the lacinia are uncovered. Orientation: a, anterior; l, lateral. lac, lacinia; lap, lapillus. Scale bars, 381 µm (a); 880 µm (b–d).

### 3.4 Hair Cell Type and Density Distributions

In all maculae, three types of hair cells are characterized (F1, F2, and F3), following the categorization of Popper (1977), which vary in their densities in different regions of the sensory epithelia (Figures 8a, 10a, and 12a).

**FIGURE 8:**
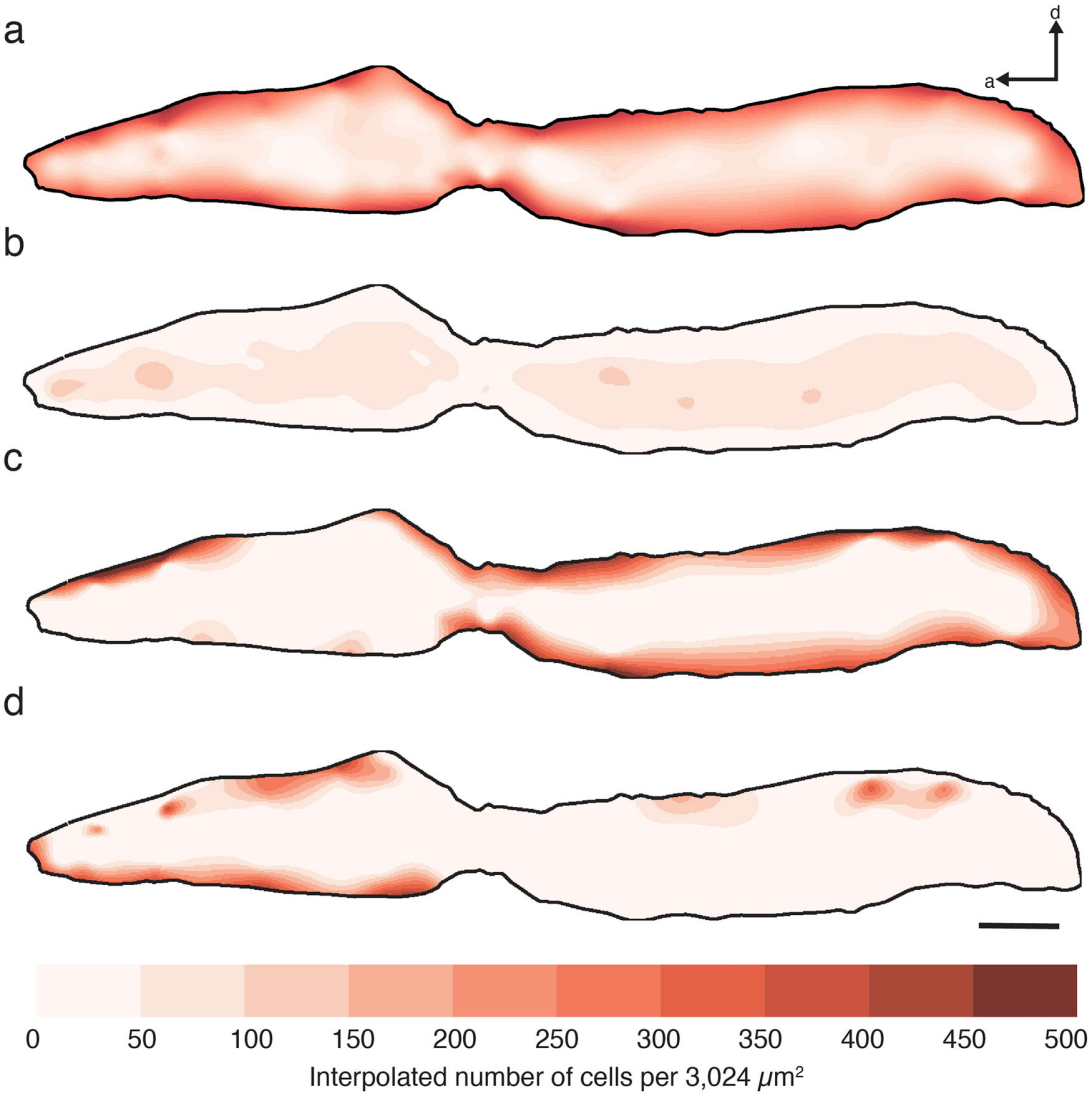
Iso‐density contour maps of hair cell density within the left saccular macula of *Thunnus maccoyii* (SBT20). (a) shows the density distribution of every hair cell bundle, while (b), (c), and (d) show the density distribution maps of F1, F2, and F3 type hair cells, respectively. Orientation: a, anterior; d, dorsal. Scale bar, 892 µm.

*Saccule*: In the saccular macula, the F1‐type hair cell bundles have the shortest kinocilium (1.8 µm in length) with short graded stereocilia and the weakest structural asymmetry (Figure 9a). They are found in low density (12,900 ± 700 mm^−2^) and exclusively occupy a wide central region of the saccular macula (Figure 8b). In total, the low‐density central region comprises about 76.6% of the macular area (Table 5). The F2‐type hair cells have the longest kinocilium (6 µm in length) with the shortest graded stereocilia, and, therefore, have the strongest structural asymmetry (Figure 9c). The F3‐type hair cells have a long kinocilium (5 µm) with long graded stereocilia comparable in length to the kinocilium (Figure 9b). The F2 and F3 types of hair cell bundles are found in high densities (45,000 ± 2,000 mm^−2^) and exclusively occupy the relatively narrow peripheral area, which extends about 500 µm from the macular boundaries (Figure 8c,d). In total, the high‐density peripheral region comprises about 23% of the epithelium (Table 5). The F2‐type hair cells are found throughout the peripheral area of the macula except for the ventral periphery of the anterior region, which is occupied by the F3‐type hair cells (Figure 8c,d). The F3 hair cells are also found in the dorsal periphery of the anterior and posterior regions, while they are not found in the periphery of the intermediate region and the ventral periphery of the posterior region (Figure 8d). In the dorsal periphery of the anterior and posterior regions, where both F2 and F3 hair cells are found, the F3 hair cells are distributed medial to the F2 hair cells. The left saccular macula of SBT19 has a total number of 195,176 hair cells with a density of 20,459 hair cell bundles per mm^2^, while the right saccular macula has a total number of 198,051 hair cells with a density of 20,717 hair cell bundles per mm^2^ (Tables 4 and 5). The left saccular macula of the SBT20 has a total of 247,803 hair cells with a density of 20,114 hair cell bundles per mm^2^, and the right saccular macula has a total of 242,367 hair cells with a density of 20,206 hair cell bundles per mm^2^ (Tables 4 and 5). Overall, the saccule of *T. maccoyii* has a mean of 220,849 hair cells and a mean hair cell density of 20,400 hair cell bundles per mm^2^, which is the lowest among the three maculae. Comparison of the saccule between SBT19 and SBT20 shows that, as *T. maccoyii* grows from 152 to 176 cm, the total number of saccular hair cell bundles increases approximately 25%, while the surface area of the macula increases approximately 27%. As the ratio between the surface area of low‐density and high‐density regions remains consistent, the hair cell density also remains unchanged (Tables 4 and 5).

**FIGURE 9:**
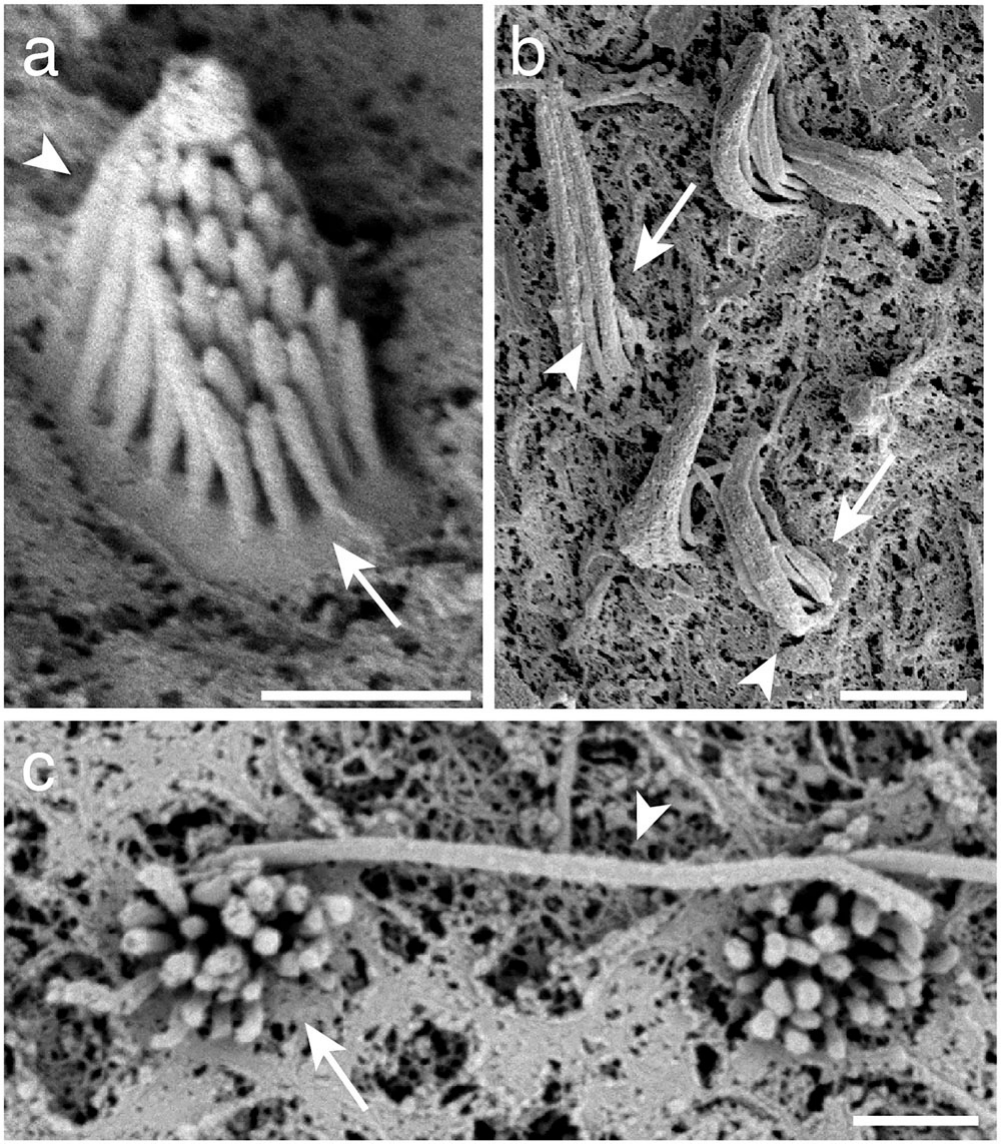
Scanning electron micrographs of different hair cell types in the saccule. Kinocilium (arrowhead) and the stereocilia bundle (arrow) are labeled. (a) F1 type hair cell. (b) F3 type hair cells. Note that several stereocilia are as long as the kinocilium. (c) F2 type hair cell. Scale bars, 2 µm (a, b); 1 µm (c).

**TABLE 5:** Quantitative assessment of the hair cell density of two mature *T. maccoyii* specimens. The maculae are divided into high‐density and low‐density regions, and the corresponding hair cell densities are provided with the total density.

		SBT19 (70 kg)		SBT20 (96 kg)		Mean ± SE
		L	R	L	R	
Saccule	High density area (%)	23	22	23	25	
	Low density area (%)	77	78	77	75	
	Hair cells mm^−2^ (high density)	40,676	45,055	47,850	46,328	45,000 ± 2000
	Hair cells mm^−2^ (low density)	14,347	13,697	11,975	11,417	12,900 ± 700
	Total density	20,459	20,717	20,114	20,206	20,400 ± 100
Lagena	High density area (%)	—	—	46	45	
	Low density area (%)	—	—	54	55	
	Hair cells mm^−2^ (high density)	—	—	49,483	49,935	49,700 ± 200
	Hair cells mm^−2^ (low density)	—	—	29,491	31,749	31,000 ± 1000
	Total density	—	—	38,695	39,940	39,300 ± 600
Utricle	High density area (%)	—	—	64	65	
	Low density area (%)	—	—	36	35	
	Hair cells mm^−2^ (high density)	—	—	43,536	44,262	43,900 ± 400
	Hair cells mm^−2^ (low density)	—	—	22,486	23,301	22,900 ± 400
	Total density	—	—	35,919	36,915	36,400 ± 500

Abbreviations: L, left ear; R, right ear; SE, standard error.

*Lagena*: In the lagenar macula, the distribution pattern of hair cell types is similar to that of the saccular macula (Figure 10b–d). The F2‐type hair cells (kinocilium length of 7–8 µm) (Figure 11a,b) and the F1‐type hair cells (kinocilium length of 3–4 µm) (Figure 11c) are found in the peripheral and the central areas, respectively, while the F3‐type hair cells (kinocilium length of 5 µm) (Figure 11d) occupy the medial area between these two zones. The ratio between the area of the high‐density (peripheral) region and the low‐density (central) region is 45:55 (Table 5). The left lagenar macula of SBT20 has a total number of 205,084 hair cells with a density of 38,695 hair cell bundles per mm^2^, and the right macula has a total number of 209,283 hair cells with a density of 39,940 hair cell bundles per mm^2^. Overall, the lagena of *T. maccoyii* has a mean of 207,184 hair cells and a mean hair cell density of 39,300 hair cell bundles per mm^2^, which is the lowest and the highest among the three maculae, respectively (Tables 4 and 5). When overlaid with the area of the lagenar macula that is covered by the asteriscus, it is primarily the hair cells with a long kinocilium (F2 and F3 types) that are uncovered by the otolith and only covered by the otolithic membrane (Figure 14a).

**FIGURE 10:**
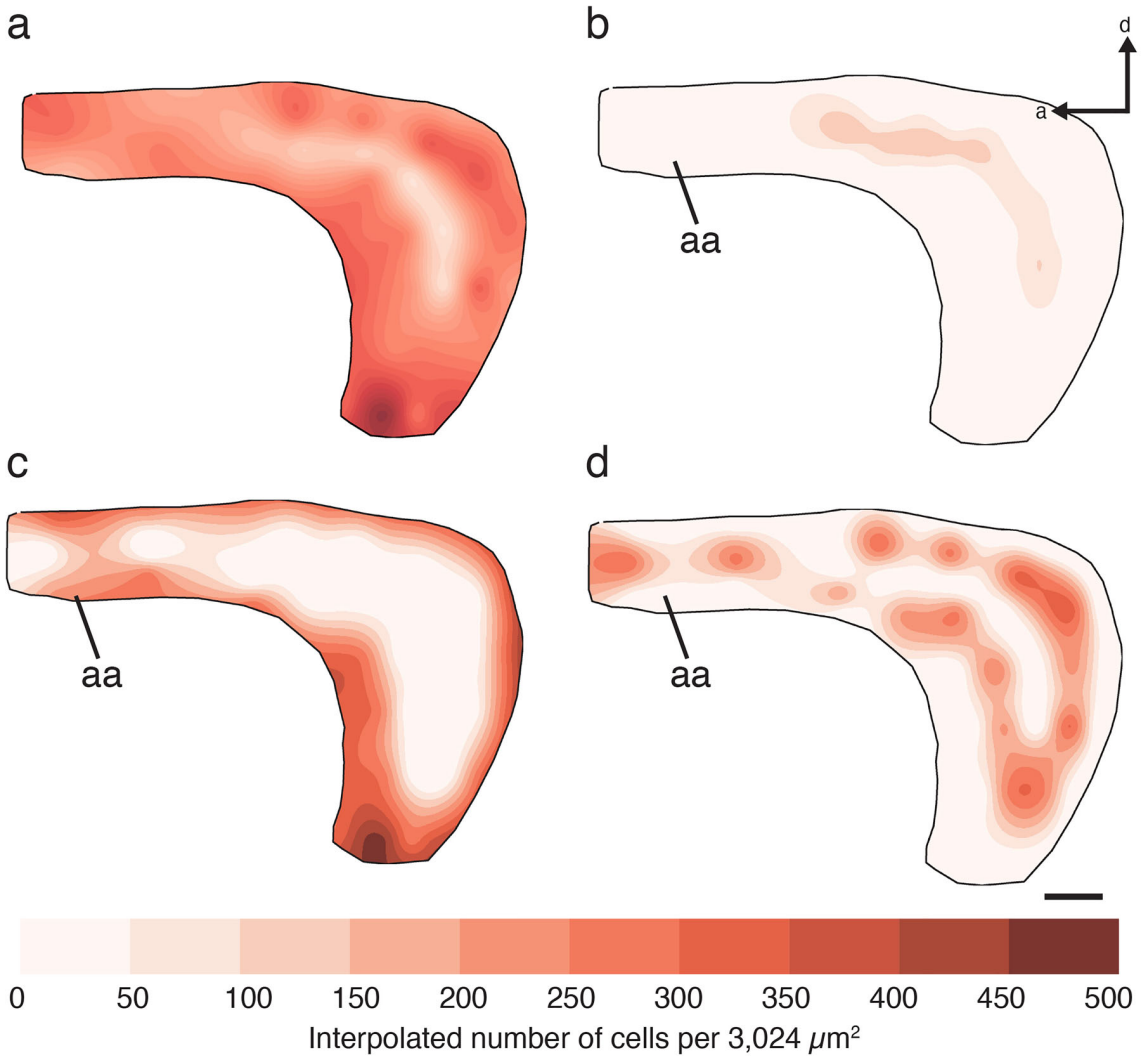
Iso‐density contour maps of hair cells within the left lagenar macula of *Thunnus maccoyii* (SBT20). (a) shows the density distribution map of every hair cell, while (b), (c), and (d) show the density distribution of F1, F2, and F3 type hair cells, respectively. Orientation: a, anterior; d, dorsal. aa, anterior arm. Scale bar, 520 µm.

**FIGURE 11:**
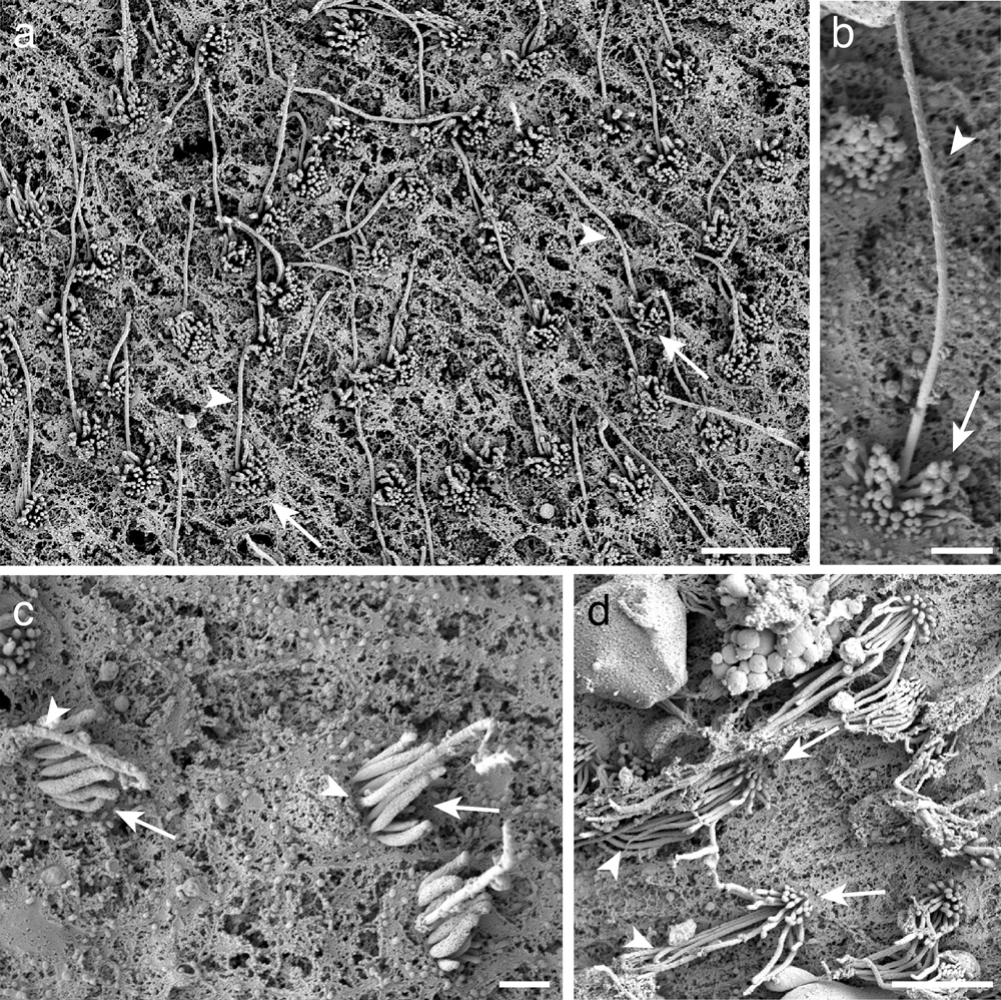
Scanning electron micrographs of different hair cell types in the lagena. Kinocilium (arrowhead) and the stereocilia bundle (arrow) are labeled. (a) High‐density group of F2 type hair cells in the periphery of the lagena. (b) Individual F2 type hair cell. (c) F1 type hair cells. (d) F3 type hair cells. Scale bars, 4 µm (a); 1 µm (b); 1 µm (c); 4 µm (d).

*Utricle*: In the utricular macula, the F2‐type hair cells (kinocilium length of 13–19 µm) (Figure 13c,d) cover the tail‐like lacinia in high density and extend anteriorly following the lateral peripheral margin (Figure 12c). The F3‐type hair cells (kinocilium length of 5–8 µm) (Figure 13e) are found medial to F2‐type hair cells and are primarily present in the lacinia and the rostro‐medial part of the macula (Figure 12d). The F1‐type hair cells (kinocilium length of 4 µm) (Figure 13a,b) occupy the central region and the medial periphery (Figure 12b). The ratio between the area of the high‐density region and the low‐density region is 64.5:35.5 (Table 5). The left utricular macula of SBT20 has a total number of 315,729 hair cells and a density of 35,919 hair cell bundles per mm^2^, while the right macula has a total number of 315,997 hair cells and a density of 36,915 hair cell bundles per mm^2^. Overall, the utricle of *T. maccoyii* has a mean of 315,863 hair cells and a mean hair cell density of 36,400 hair cell bundles per mm^2^ (Tables 4 and 5). The lapillus primarily covers the short F1‐type hair cells on the medial region of the utricular macula, leaving the long F2‐ and F3‐type hair cells in the lacinia and lateral periphery mostly uncovered (Figure 14b). All three maculae show strong left and right symmetry in terms of both hair cell number and orientation.

**FIGURE 12:**
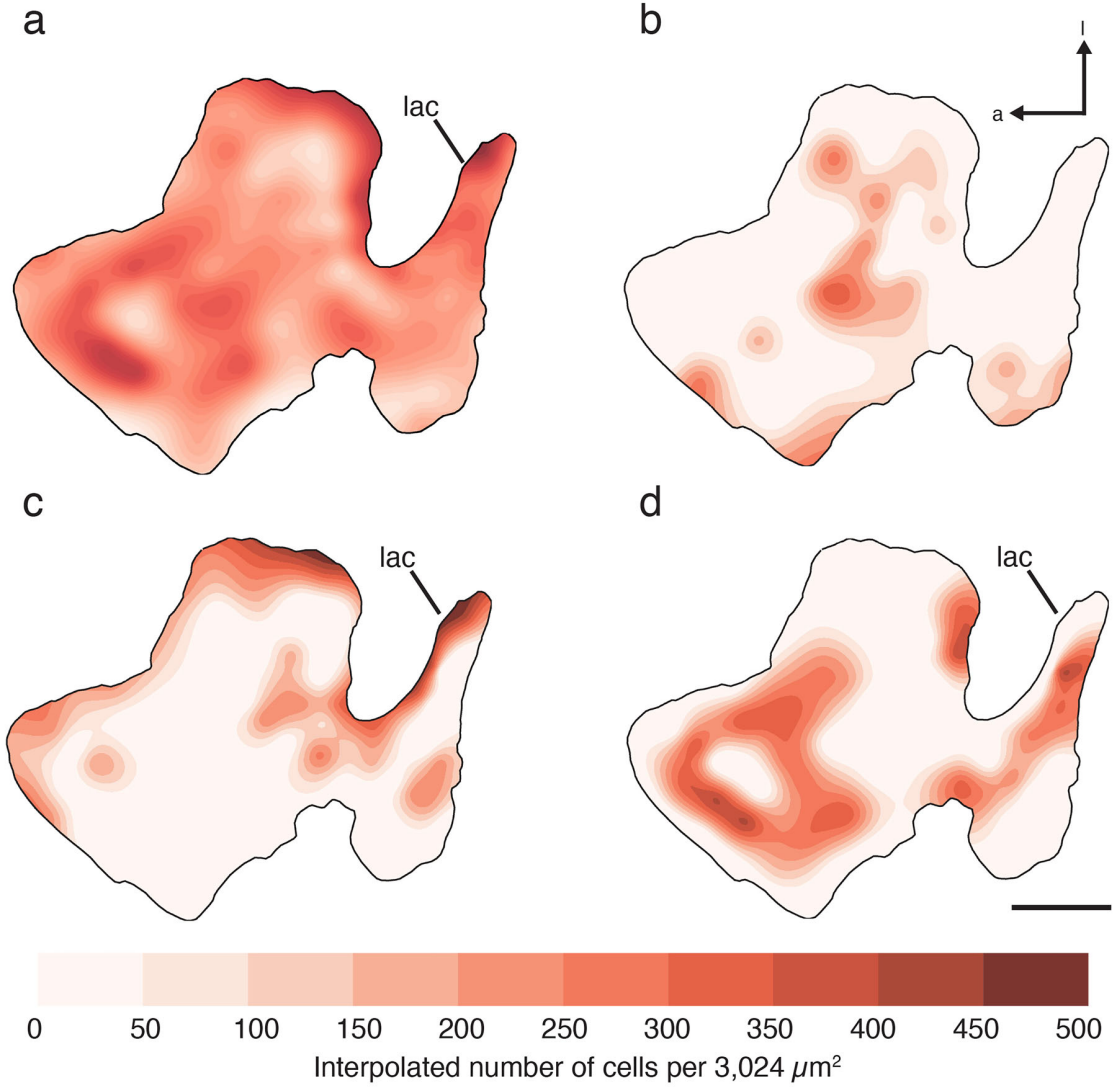
Iso‐density contour maps of hair cells within the right utricular macula of *Thunnus maccoyii* (SBT20). (a) shows the density distribution of every hair cell, while (b), (c), and (d) show the density distribution of F1, F2, and F3 type hair cells, respectively. Orientation: a, anterior; l, lateral. lac, lacinia. Scale bar, 880 µm.

**FIGURE 13:**
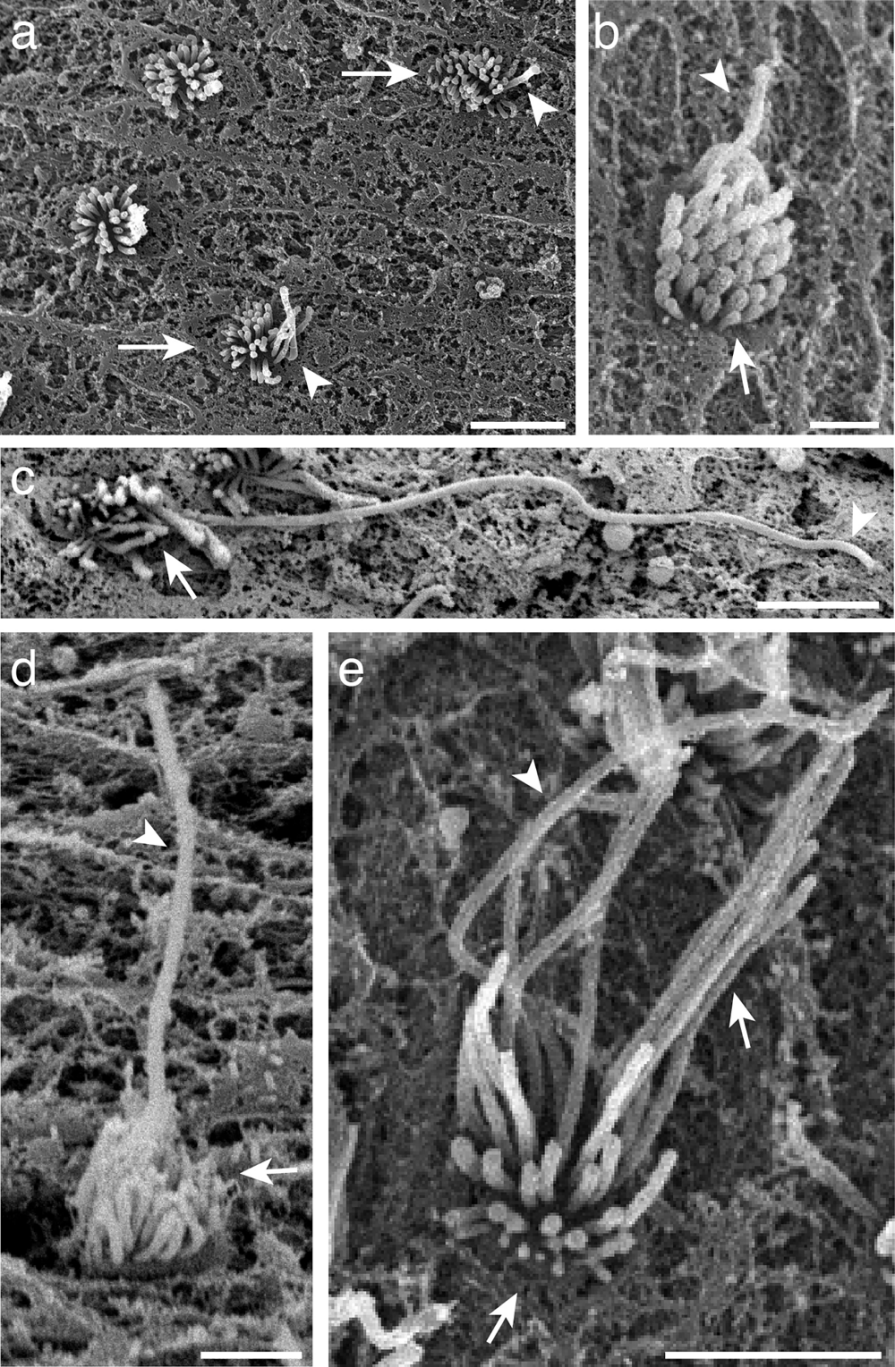
**|** Scanning electron micrographs of different hair cell types in the utricle. Kinocilium (arrowhead) and the stereocilia bundle (arrow) are labeled. (a) F1‐type hair cells. (b) F1‐type hair cell. (c) F2‐type hair cell in a lacinia. Note that the length of the kinocilium is around 20 µm. (d) F2‐type hair cell in the anterior periphery of the utricle. Note that the length of the kinocilium is much shorter compared to the F2‐type hair cell in lacinia. (e) F3‐type hair cell. Scale bars, 2 µm (a); 1 µm (b); 2 µm (c); 1 µm (d); 2 µm (e).

**FIGURE 14:**
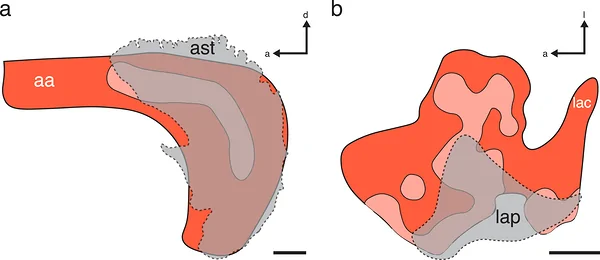
**|** Schematic illustrations of the distribution of hair cells with short kinocilia (F1‐type, in light red) and long kinocilia (F2‐ and F3‐types in dark red) on the otolith‐covered lagena and utricle of *Thunnus maccoyii*. (a) The asteriscus primarily covers the short hair cell type, and the long hair cell type on the anterior arm of the lagena is uncovered. (b) The lapillus primarily covers the short hair cell type, and the long hair cell type on the lacinia of the utricle is uncovered. Orientation: a, anterior; d, dorsal; l, lateral. aa, anterior arm; ast, asteriscus; lac, lacinia; lap, lapillus. Scale bars, 520 µm (a); 880 µm (b).

## 4 Discussion

This is the first study to examine the morphology of the inner ear of southern bluefin tuna *T. maccoyii*. Some morphological characteristics of the semicircular canals and end organs, the rostro‐caudal connection between the saccule and lagena, the relative size of the three end organs, and the hair cell orientation patterns in the lagena and the utricle, appear to be similar to those of the Atlantic bluefin tuna *T. thynnu*s and the skipjack tuna *K. pelamis* (Popper, 1981; Song et al. 2006). However, several morphological features are species‐specific, including the relative area of the otolithic membrane covered by the otolith in both the lagena and the utricle and the unique saccular hair cell orientation pattern with three distinct sets of opposing hair cell orientations (Popper, 1981; Song et al. 2006).

### 4.1 The Gross Morphology of the Inner Ears

The inner ears of *T. maccoyii* are comparatively large, with their height (∼12 mm) protruding above the height of the brain and their length (∼ 17 mm) comparable to the length of the brain in a 152 cm (*L*_F_) individual (Figure 1c). Such spatial dimensions have notably been found for the inner ears in some deep‐sea fishes, which seem to occupy a relatively larger space than the brain within the cranium (Deng et al. 2011, 2013, 2023). Both upper and lower components of the inner ears in *T. maccoyii* are tightly enclosed within the cranium, with little to no space between the membranous wall and the surrounding otic capsules. The membranous walls of the semicircular canals and end organs of *T. maccoyii* were also found to be thick and cartilage‐like, similar to *T. thynnus* and the deep‐sea teleost *Antimora rostrata* (Song et al. 2006; Deng et al. 2011). As suggested by Song et al. (2006), this tight arrangement likely stabilizes and protects the inner ear labyrinths within the cranium, reducing any movement produced by the inertial force generated from this species’ propensity for linear acceleration and abrupt turns. Tight envelopment by the otic capsule also ensures that hair cells in the maculae and ampullae are only stimulated by the motion of the otoliths and cupulae, rather than by the movements of inner ear parts inside the cranium, which could introduce noise into vestibular and auditory processing (Hawkins, 1986). As these features were also found in the inner ears of *T. thynnus*, it is possible that such morphological characteristics may have evolved in large pelagic fishes as a whole in order to provide stability and protection during acceleration (Song et al. 2006). Further examination of other large pelagic species, including non‐tuna species, is necessary to confirm this hypothesis.

### 4.2 The Structure and Function of the Otolithic Membranes

A translucent jelly‐like material was identified on the medial surface of every otolith (sagitta, asteriscus, and lapillus). In the sagitta, the jelly‐like material is perfectly molded to the sulcus. Similar structures have been identified on the medial surface of the otoliths of a few other species of teleosts: Atlantic bluefin tuna *T. thynnus*, a number of species within the family Chaetodontidae (*Chaetodon auriga*, *C*. *multicinctus*, and *Forcipiger flavissimus*), and the morid cod *A. rostrata* (Song et al. 2006; Webb et al. 2010; Deng et al. 2011). In *A. rostrata*, the material was identified as the otolithic membrane—a mesh‐like structure mediating the interface between the otolith and the sensory hair cells (Deng et al. 2011). However, in other species, the identity of this jelly‐like material remains uncertain. The analysis of the jelly‐like material in this study using SEM reveals a porous, mesh‐like surface morphology with hair cells embedded within the pores, confirming the identity of the material as the otolithic membrane in *T. maccoyii*. Furthermore, subsequent investigation found that the shape of this gelatinous structure matched exactly the shape of the underlying macula. The exact function of the otolithic membrane remains unclear, although it is suggested to affect how hair cells respond to different elements of sound particle motion by rendering the coupling between the otolith and the hair cells elastic (Kalmijn, 1988; Rogers and Cox, 1988). In *T. maccoyii*, the otolithic membranes in the lagena and the utricle completely cover the maculae, including the regions that are not overlain by the otoliths, which may provide the structural basis for the multipole mechanisms proposed for resolving the 180° ambiguity (Rogers and Zeddies, 2008). Similar morphological characteristics can also be found in the lagena of *A. rostrata* and *C. auriga* (Webb et al. 2010; Deng et al. 2011).

On the sagitta of the largest *T. maccoyii* examined (190 cm *L*_F_, 122 kg), jelly‐like material was also found on the lateral surface, indicating it cannot be an otolithic membrane (Figure 15). Interestingly, the “lateral jelly” was also found on the sagitta of *T. thynnus*, and it contained a set of “growth rings” similar to those found on the sagitta of teleosts (Song et al. 2006). The growth rings were not found in the lateral jelly of *T. maccoyii*. Since the lateral jelly was only discovered in the largest individual and the *T. thynnus* specimens examined by Song et al. (2006) were even larger, the formation of the lateral jelly may only occur in large mature fishes. A greater number of sagittas of large *T. maccoyii* must be investigated to resolve whether the growth rings are a shared feature among large scombrids. The lateral jelly is hypothesized to stabilize the position of the otoliths in the end organ chamber (Song et al. 2006), which may be more relevant to larger individuals.

**FIGURE 15:**
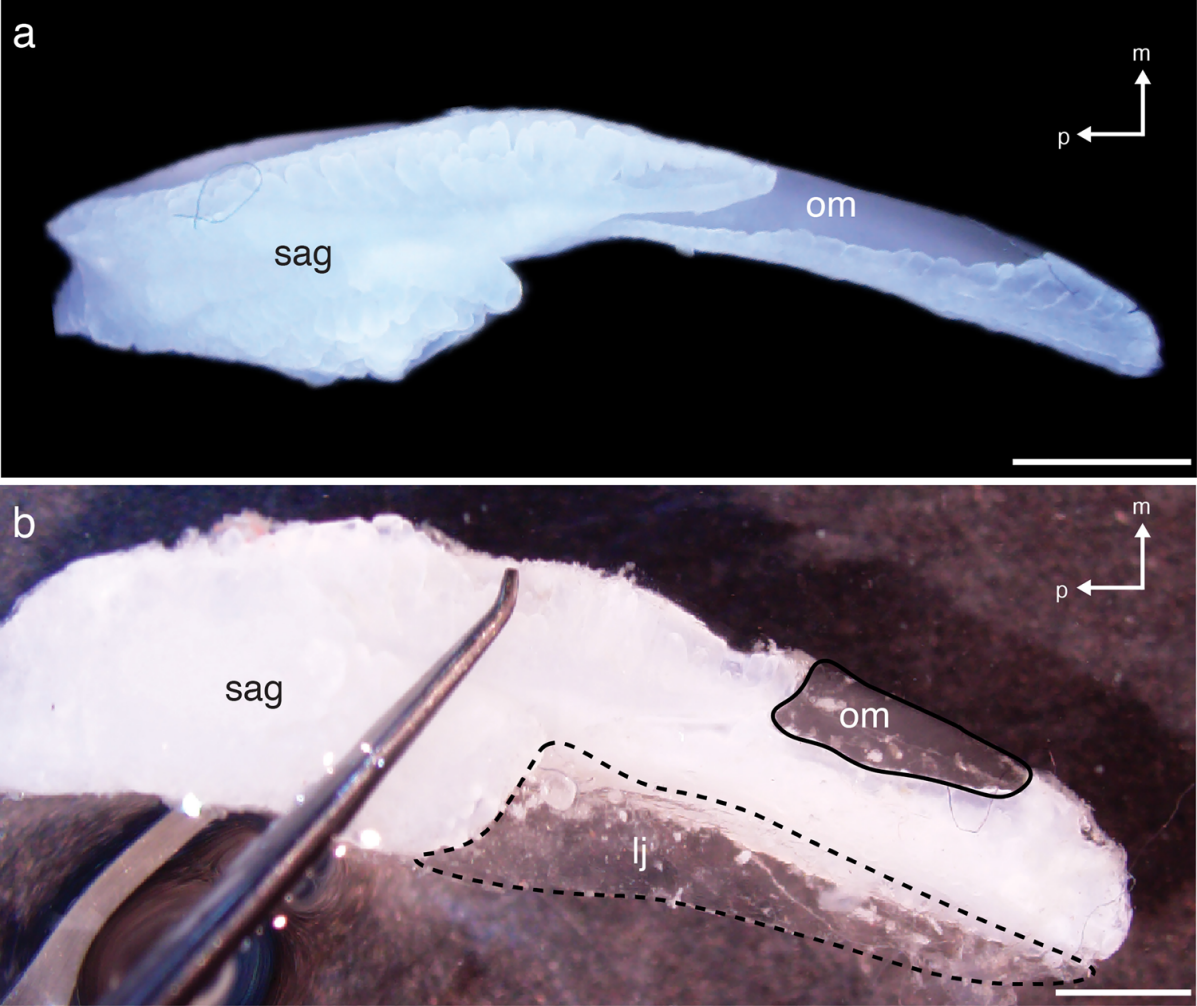
**|** Photographs of the sagitta (sag) of a 70 kg (a) and 122 kg (b) *Thunnus maccoyii* in dorsal view. The otolithic membrane (om) fills up the sulcus on the medial surface of the sagitta in both specimens. The 122 kg specimen has a translucent jelly‐like material (lateral jelly, lj) on the lateral surface of the sagitta as well. Orientation: m, medial; p, posterior. Scale bars, 2 mm (a, b).

### 4.3 Hair Cell Types and Orientation in Three Maculae

The elongated shape and distribution pattern of hair cell type and density of the saccular macula of *T. maccoyii* are very similar to those of *T. thynnus* and *K. pelamis* (Popper, 1981; Song et al. 2006). The peripheral areas are occupied by the F2‐ and F3‐type hair cells in high density, while the central region is occupied by the F1‐type hair cells in low density. The hair cell orientation pattern of the saccule of *T. maccoyii* is also mostly similar to the other species of tunas. The saccules of *T. thynnus* and *K. pelamis* have hair cells organized into four quadrants (i.e. standard pattern), where two horizontal groups are at the rostral part of the macula, while two diagonal groups are at the caudal part (Popper, 1981; Song et al. 2006). This is slightly different from the typical “standard” pattern commonly found among teleosts, which have two vertical groups at the caudal part (Popper and Coombs, 1982; Schellart and Popper, 1992). In *T. maccoyii*, the rostral and caudal parts of the saccular macula, each of which coincides with the sulcus ostium and cauda, respectively, have two horizontal and two diagonal groups of hair cells. However, *T. maccoyii* possesses an additional, small intermediate zone, which coincides with the collum of sulcus, and is occupied by two groups of vertically oriented hair cells. There are, therefore, three groups of hair cells of opposing orientation in the saccule of *T. maccoyii*. Among the three opposing orientation groups, the diagonally‐oriented hair cells occupy the largest area and are the most abundant in *T. maccoyii*.

The “dual” and “alternating” patterns, which also have three groups of hair cells of opposing orientation, have two sets of horizontal groups, which share the same orientation axis, and one set of vertical groups (Popper et al. 2003; Ladich and Schulz‐Mirbach, 2016). In comparison, the orientation axis of each opposing group in the saccule of *T. maccoyii* does not overlap, where the hair cells in the rostral part would best respond to sound particle motion along the horizontal axis, while the hair cells in the caudal part best respond to sound particle motion along the diagonal axis, and the hair cells in the intermediate part best respond to sound particle motion along the vertical axis. Hence, the saccular hair cell orientation pattern of *T. maccoyii*, which we describe as “triaxial,” has three opposing hair cell groups, each with its own distinctive axis of sensitivity. Due to the relatively smaller macular area and hair cell numbers, this intermediate area may be a transition zone between the rostral and caudal areas. However, a distinct branch of the VIIIth cranial nerve specifically innervates the intermediate region of the sacculus. It has been demonstrated that the regions of saccular maculae having different hair cell orientations are innervated by a separate branch of the eighth cranial nerve in several teleosts (Saidel and Popper, 1983; Deng et al. 2013). In addition, the thickness of each saccular nerve branch or ramus of *T. maccoyii*, innervating a specific orientation cell group, appears to be proportional to both hair cell number and the area of the macula occupied by that specific orientation group. Since the VIIIth nerve branch innervating the intermediate region is significantly thinner compared to the other nerve branches, it is highly likely that this ramus is dedicated to processing sound detected at this locus.

This additional orientation group will likely add precision to the directional hearing ability of the inner ears in *T. maccoyii*. Since the three groups of hair cells with opposing orientation are not overlapping in their orientation and each is innervated by a separate branch of the VIIIth nerve, three independent channels (vertical, horizontal, and diagonal) exist in the saccule of *T. maccoyii* in contrast to most teleosts, which possess only the vertical and horizontal channels (Popper et al. 1988; Rogers et al. 1988; Schellart and Popper, 1992). Therefore, the saccule of *T. maccoyii* may compute the angle of incidence of an acoustic signal by weighting three unique vector responses instead of two, which we hypothesize will enhance the precision of the calculated angle, thus providing a significant advantage in tracking prey in featureless open water habitats (Popper et al. 1988; Rogers et al. 1988). Future work should quantify the number of hair cells innervated by each nerve branch of the VIIIth nerve to assess the level of directional precision mediated by the saccule in *T. maccoyii*.

In the lagena of *T. maccoyii*, the long F2‐ and F3‐type hair cells occupy the marginal region, including the rostral and ventral ends of the macula, while the short F1‐type hair cells occupy the narrow area along the center. In the utricle, the distribution is similar in having the long F2‐ and F3‐type hair cells around the periphery and the short F1‐type hair cells in the central region, although the F1‐type hair cells are also found in the temporal margin. It is interesting to note that while the distribution of hair cell types in the lagena and the utricle of *T. maccoyii* is similar to that of *T. thynnus* (Song et al. 2006), the lagena of *K. pelamis* does not possess F1‐ or F2‐type hair cells but only possesses F3‐type hair cells (Popper 1981). Examination of the lagena and utricle of other tuna species within the tribe Thunnini, comprising five genera, is crucial to understanding the degree of interspecific variation in hair cell types.

Both the lagena and utricle of *T. maccoyii* possess one opposing pair of hair cell orientation groups, which are parallel to each other in the lagena and facing each other in the utricle. The line dividing the orientation groups is curved and follows the contour of the maculae, and the hair cell orientation curves accordingly, essentially covering all horizontal, vertical, and diagonal axes. The hair cell orientation pattern of the lagena and utricle appears to be strongly conserved among teleosts, and the pattern in *T. maccoyii* is very similar to *T. thynnus* and *K. pelamis* (Popper, 1981; Popper et al. 2003; Song et al. 2006; Ladich and Schulz‐Mirbach, 2016). Elasmobranchs also possess hair cell groups with opposing orientations in their lagena, but the angle of orientation does not follow its horseshoe shape, strictly maintaining a dorso‐ventral orientation, thereby emphasizing the significance of detecting sound coming from above and below for this taxon (Sauer et al. 2023).

### 4.4 Otolith Coverage and Solving the 180° Ambiguity

The otolithic membrane and maculae of the lagena and utricle in *T. maccoyii* are only partially covered by the otolith. This curious mismatch is also found in a diversity of other teleost species living in varying habitats, ranging from the deep‐sea (*Coryphaenoides guentheri*, *Coryphaenoides rupestris*, *Nezumia aequalis*, etc.), to pelagic (*T. thynnus*), to shallow‐water (*Carassius auratus* and *C. auriga*), although the proportion of macular area covered by the otolith appears to vary (Platt, 1977; Popper, 1980; Song et al. 2006; Webb et al. 2010; Deng et al. 2011, 2023). The relative area of the macula covered and uncovered by the otolith has rarely been a focus when investigating the morphological characteristics of the inner ear, where the otolith coverage has mostly been described qualitatively (although see Deng et al. 2023). Therefore, it is difficult to quantitatively compare the level of otolith coverage between species and the impact this may have on auditory processing.

It appears that the otolith coverage of the lagena of *T. maccoyii* (72%) is similar to that of macrourid fishes (e.g., *C. rupestris*: 65%; *N. aequalis*: 78%) and a pebbled butterflyfish (*Chaetodon multicinctus*: 78%) (Webb et al. 2010; Deng et al. 2023), while the lagenar otolith of *Coryphaenoides guentheri*, a bathydemersal teleost, only covers 18% of the macula (Deng et al. 2023). Based on the qualitative description, the coverage of the lagenar and utricular otoliths of the goldfish *C. auratus* appears greater than that of *T. maccoyii* (Platt, 1977). In species with partial otolith coverage, the uncovered regions, such as the lacinia of the utricle and the anterior tip of the lagena, are consistently populated by hair cells with long kinocilium (Platt, 1977; Webb et al. 2010; Deng et al. 2011, 2023). These hair cells are hypothesized to function as quadrupole sensors, responding to fluid shear induced by sound particle motion at approximately 45° to the anatomical axes (Rogers and Cox, 1988; Rogers and Zeddies, 2008). Unlike dipole sensors that rely on otolith‐induced shearing of the ciliary bundle, these uncovered hair cells are hypothesized to be deflected directly by fluid motion (Rogers and Zeddies, 2008). As a result, their activation likely requires higher stimulus thresholds. This may explain the placement of long‐kinocilium hair cells in regions not overlain by otoliths. Further comparative studies across teleosts occupying diverse habitats are needed to assess the functional role and distribution of hair cell types of these uncovered sensory regions.

The presence of uncovered, off‐axis hair cells may help resolve the 180° directional ambiguity typical of dipole sensors—particularly in species without a swim bladder or with a weak connection between the swim bladder and the inner ear—by providing directionally‐distinct input (Rogers and Zeddies, 2008). By combining the inputs of both covered (dipole‐like) and uncovered (quadrupole‐like) hair cells, the system may resolve the 180° directional ambiguity and achieves greater directional resolution than that of a monopole model (i.e., which involves a swim bladder) enabling more precise localization of sound sources (Rogers and Zeddies, 2008). Notably, the sharper directivity provided by quadrupole sensors improves the signal‐to‐noise ratio by approximately 3.4 dB (i.e., more than a twofold enhancement) over monopole sensitivity (Popper et al. 1988; Rogers and Zeddies, 2008). Furthermore, while uncovered hair cells respond specifically to sound‐induced shear strain, otolith‐covered hair cells are broadly sensitive to whole‐body acceleration, including non‐acoustic sources of motion (Rogers and Zeddies, 2008). Thus, the partial otolith coverage observed in *T. maccoyii* may represent an adaptive feature for resolving the 180° ambiguity and improving sound detection in teleosts that lack swimbladder or whose swimbladder does not contribute acoustically (Rogers and Zeddies, 2008).

### 4.5 Hair Cell Density and Implications for Audition

Comparing hair cell density and total hair cell number across teleost species remains challenging, not only because relatively few studies have quantified these parameters, often focusing solely on the saccule, but also because even fewer have reported total hair cell abundance across the entire sensory epithelium using standardized methods, such as the protocol established by Popper and Hoxter (1984). Due to the differential density of hair cells across the macula, the total number of hair cells can be accurately estimated only when hair cell numbers are separately assessed in both high‐ and low‐density areas and summed.

The mean hair cell number of the saccule of *T. maccoyii* is about 220,849, which is substantially less than that of *T. thynnus* (1,151,660). This is not surprising, as specimens of *T. thynnus* investigated by Song et al. (2006) were more than three times heavier than the *T. maccoyii* used in this study and had significantly larger macular surface areas (∼38 mm^2^) in the saccule. European hake (*Merluccius merluccius*) also has considerably more hair cells (∼700,000) than *T. maccoyii*, despite being much smaller in body size (Lombarte and Popper, 1994). However, the saccular surface area of *M. merluccius* (∼35 mm^2^), which is comparable to *T. thynnus*, is substantially larger than that of *T. maccoyii* (∼11 mm^2^). On the other hand, *T. maccoyii* has a higher number of hair cells compared to teleost species with smaller saccular macula areas (Table 6).

**TABLE 6:** Differences in the area, number of hair cells, and density range of hair cells in the saccular maculae of different teleost species.

Species	Area (mm^2^)	Number	Density range (2500 µm^−2^)	References
*Thunnus maccoyii*	10.9	220,849	32–112	This study
*Thunnus thynnus*	38.5	1,151,660	63–105	Song et al. 2006
*Scomber japonicus*	—	—	37–111	Smith et al. 2022
*Merluccius merluccius*	35	∼700,000	37.5–60	Lombarte and Popper 1994
*Gadus morhua*	—	—	∼62	Faucher et al. 2009
*Astronotus ocellatus*	0.6	32,139	98–171	Popper and Hoxter 1984
*Carassius auratus*	0.3^a^	20,000–50,000	50–125	Platt 1977
*Chaetodon auriga*	1.1	24,710–48,290	55–107.5	Webb et al. 2010
*Chaetodon multicinctus*	0.6	13,790–33,860	55–135	Webb et al. 2010
*Chaetodon ornatissimus*	0.5	17,250–39,890	80–185	Webb et al. 2010
*Forcipiger flavissimus*	1.0	22,470–52,760	57.5–135	Webb et al. 2010

^a^Values measured from the published figures.

This implies that there is a positive correlation between hair cell number and the macular area in the saccule. The hair cell numbers within the saccule of *T. maccoyii* appear to increase with body size and macula area, which is consistent with previous reports on the correlative relationship between fish length and saccule area and hair cell number (Popper and Hoxter, 1984; Lombarte and Popper, 1994; Song et al. 2006). The hair cell densities within the lagena and utricle are similar in *T. maccoyii* and *T. thynnus*, while *T. thynnus* has a higher density of hair cell bundles in the saccule (Table 7) (Song et al. 2006). The small differences in hair cell density in the lagena and utricle in the two species can likely be attributed to variability in the sample size and differences in demarcating the low and high density areas. The large interspecific difference in the density of hair cells within the saccule is due to relatively higher hair cell densities over a large proportion of the macula in *T. thynnus* (Table 7). When compared to the hair cell density of the saccule in the chub mackerel *Scomber japonicus* (Smith et al. 2022), the only other scombrid species where hair cell density data are available, *T. maccoyii* and *S. japonicus* have similar hair cell densities (Table 6).

**TABLE 7:** Differences in the average hair cell density (mm^−2^) between *Thunnus maccoyii* (this study) and *Thunnus thynnus* (Song et al. 2006). Differences in low‐density and high‐density regions are included. * Recalculated from Tables I and II in Song et al. (2006). The pinted value (32,429) in Table II of Song et al. (2006) duplicates their low‐density region value and is inconsistent with their regional data.

		*Thunnus maccoyii* (*n* = 4 (saccule, 2 (lagena, utricle))	*Thunnus thynnus* (*n* = 5)
Sac	Total	20,400	29,915
	Low	12,900	25,309
	High	45,000	42,052
Lag	Total	39,300	34,469
	Low	31,000	29,342
	High	49,700	41,793
Utr	Total	36,400	42,579^*^
	Low	22,900	32,429
	High	43,900	47,128

Abbreviations: Lag, lagena; Sac, saccule; Utr, utricle.

While the number of hair cells has shown positive correlation with hearing sensitivity in a couple of teleost species (Coffin et al. 2012; Wang et al. 2015), the results are from “hearing specialists” and vocal teleosts. It is, hence, difficult to predict the hearing abilities of *T. maccoyii* and other tunas simply based on the number and density of hair cells, as very little is known on the role of the swimbladder in hearing of tunas (Gibbs and Collette, 1967; Schaefer and Oliver 2000). Tunas with (*T. orientalis* and *T. albacares*) and without (*E. affinis*) a swimbladder showed a similar hearing range and frequency of greatest sensitivity (*f_b_*: 400–500 Hz) (Iversen, 1967, 1969; Dale et al. 2015). Tunas with a swimbladder showed a lower hearing threshold at *f_b_*, which suggests a potential role of the swimbladder in enhancing hearing sensitivity. However, there is no evidence of any close connection between the inner ears and swimbladder in *T. orientalis* or *T. albacares*. Hair cell numbers have not been quantified in tunas (*T. albacares*, *T. orientalis*, and *E. affinis*) whose hearing sensitivity was measured (Iversen, 1967, 1969; Dale et al. 2015), and, on the other hand, hearing sensitivity has not been measured in tunas (*T. thynnus* and *T. maccoyii*) whose hair cells were quantified (Song et al. 2006; this study). Due to this mismatch, the role of hair cell density in hearing range and sensitivity is difficult to speculate. To clarify this relationship, conducting both morphological and behavioral/electrophysiological investigations on the same species is critical.

## 5 Conclusions

This study provides the first detailed morphometric analysis of the inner ear in the southern bluefin tuna, *T. maccoyii*, including gross morphological analysis of the otic chamber, semicircular canals, otoliths, and otolithic membrane and quantification of hair cell types, distribution, and densities in all three otolithic end organs. The compact otic chamber encapsulating the membranous otolithic end organs and thick cartilage‐like semicircular canals may provide structural stability against frequent acceleration and turns associated with the obligate swimming lifestyle of tunas. In addition to the horizontal and diagonal hair cell orientations in the rostral and caudal parts of the saccule, respectively, vertical hair cell orientation groups are discovered in the intermediate part of the saccule, and all three groups of hair cells are innervated by separate branches of the VIII cranial nerve. This may improve the precision of vector weighting of incident particle motion, and thereby directional hearing ability, which is important in featureless pelagic environments. The maculae of lagena and utricle are only partially covered by the otoliths, and the uncovered areas are primarily occupied by hair cells with long kinocilia. Via a quadrupole mechanism, these uncovered hair cells may contribute to resolving the 180° ambiguity without the involvement of a swimbladder and improve the signal‐to‐noise ratio of sound detection. *T. maccoyii* has a large saccular macula area and high numbers of hair cells, but its functional implication is difficult to predict due to a lack of information on the hearing range and hearing sensitivity of *T. maccoyii*. Both morphological and behavioral/electrophysiological approaches must be undertaken on more species of tunas and other pelagic teleosts to further elucidate the morphological adaptations of inner ears associated with a pelagic mode of life.

## Author Contributions

**Myoung‐Hoon Ha**: conceptualization, resources, funding acquisition, methodology, investigation, data curation, formal analysis, visualization, writing – original draft, writing – review and editing. **Lucille Chapuis**: conceptualization, methodology, software, project administration, supervision, validation, writing – review and editing. **Travis L. Dutka**: conceptualization, resources, project administration, supervision, writing – review and editing. **Julian Pepperell**: conceptualization, project administration, supervision, writing – review and editing. **Caroline C. Kerr**: conceptualization, project administration, funding acquisition, writing – review and editing. **Rebecca Glarin**: methodology. **Bradford Moffat**: methodology. **Shaun P. Collin**: conceptualization, project administration, supervision, funding acquisition, validation, writing – review and editing.

## Ethics Statement

As this study used animals harvested and donated by recreational fishermen under Victorian Fisheries Authority research permit RP1447, no ethical approval was required.

## Conflicts of Interest

The authors declare no conflicts of interest.

## Data Availability

The data that support the findings of this study are available from the corresponding author upon reasonable request.
